# Conceptualizing end-of-life communication by nursing staff as part of advance care planning with older people: A multiple discipline focus group study

**DOI:** 10.1016/j.ijnsa.2025.100436

**Published:** 2025-10-25

**Authors:** Fran B.A.L. Peerboom, Jolanda H.H.M. Friesen-Storms, Jenny T. van der Steen, Daisy J.A. Janssen, Judith M.M. Meijers

**Affiliations:** a Zuyderland Medical Center, Dr. H. van der Hoffplein 1, 6162 BG Sittard-Geleen, The Netherlands; b Department of Health Services Research, Maastricht University, Care and Public Health Research Institute, Duboisdomein 30, 6229 GT Maastricht, The Netherlands; c Research Center for Autonomy and Participation in Health Care and Social Welfare and Academy of Nursing, Zuyd Health, Zuyd University of Applied Sciences, Nieuw Eyckholt 300, 6419 DJ Heerlen, The Netherlands; d Department of Public Health and Primary Care (PHEG), Leiden University Medical Center, Leiden, the Netherlands; e Radboudumc Alzheimer Center and Department of Primary and Community Care, Radboud university medical center, Nijmegen, The Netherlands; f Cicely Saunders Institute, King’s College London, UK; g Living Lab in Ageing and Long-Term Care, Maastricht University, Duboisdomein 30, 6229 GT Maastricht, The Netherlands; h Department of Expertise and Treatment, Proteion, Heythuyserweg 21, 6085 NH Horn, The Netherlands; i Department of Family Medicine, Care and Public Health Research Institute, Faculty of Health Medicine and Life Sciences, Maastricht University, Maastricht, The Netherlands; j Research Center for Nursing Leadership in a Changing Context and Research Center for Community Care, Academy of Nursing, Zuyd University of Applied Sciences, Nieuw Eyckholt 300, 6419 DJ Heerlen, The Netherlands

**Keywords:** Advance care planning, End-of-life communication, Family caregivers, Focus groups, Nursing staff, Older people, Physicians, Spiritual caregivers

## Abstract

**Background:**

Understanding the fundamentals of end-of-life communication by nursing staff as part of advance care planning with older people and their family caregivers can enhance nurse education, research, and practice in end-of-life communication. We developed a preliminary theoretical framework based on previous studies, incorporating literature and perspectives from nursing staff, older people, and family caregivers. Reflection and discussion from these groups— as well as from physicians and spiritual caregivers with whom nursing staff most often collaborate in advance care planning—should be included to ensure accuracy and enrich the framework.

**Objective:**

To enrich and provide a multiple discipline perspective on a preliminary theoretical framework for end-of-life communication by nursing staff as part of advance care planning with older people.

**Methods:**

Five semi-structured focus group interviews were conducted. Four focus groups were discipline-specific, and one included various disciplines. Data were analyzed using framework analysis; the five overarching themes and twenty fundamentals from the preliminary theoretical framework served as the coding structure.

**Results:**

Ten nursing staff members, eight physicians, seven spiritual caregivers, and eight patient-family caregiver representatives participated. The interview results enriched the framework and understanding of end-of-life communication. Participants emphasized the importance of prioritizing mutual trust over a long-term relationship, distinguishing between feeling able and being able to talk about the end of life, not having an intended outcome for the conversation other than seeking connection, and prioritizing silence over speaking to ensure that older people and family caregivers feel seen and heard.

**Conclusion:**

Nursing staff, typically very proactive professionals, may need to step back, reflect, remain silent, and sit down to connect with older people and family caregivers. The revised theoretical framework can help nursing staff become aware of, learn from, prepare for, engage in, and evaluate person-centered end-of-life communication that respects the values, needs, and preferences of the older person, their family caregivers, and themselves.

**What is already known**

Nurses are ideally positioned to play a central role in end-of-life communication.

EOL communication enables older people to play a proactive role in decision-making.

**What this paper adds**

Mutual trust may be at least as important as a long-term relationship in advanced care planning.

Nurses should aim to connect and let go of a specific outcome in end-of-life communication.

Silence may be more important than speaking in end-of-life communication.

Alt-text: Unlabelled box

## 1 Background

Timely end-of-life communication as part of advance care planning enables older people to play a proactive role in decision-making about their future end-of-life care. It may support them in receiving physical, social, spiritual, and psychological end-of-life care that aligns with their needs and preferences (Detering et al., 2010, Malhotra et al., 2022, Rietze and Stajduhar, 2015). End-of-life communication part of advance care planning with older people involves proactive, timely, informal and formal conversations between the older person, their family caregiver, and a healthcare professional. Conversations may discuss future end-of-life care, the transition to the end-of-life phase, and death and dying from a holistic perspective (Schüttengruber et al., 2022, Sudore et al., 2017, Rietjens et al., 2017). This includes discussing values; preferences regarding life-sustaining treatments and palliative care; and any discomfort, anxiety, or fear of illness and dying (McMahan et al., 2013, McMahan et al., 2021). Informal end-of-life conversations generally focus on exploring and identifying needs; these conversations may alternate with formal conversations, which focus on weighing options and making decisions (Schreiber et al., 2017).

Nursing staff are ideally positioned to play a central role in end-of-life communication with older people and their family caregivers, exploring their feelings, thoughts, and values regarding the end of life and end-of-life care. They are trained to adopt a holistic view of care and build trusting relationships with older people and their family caregivers. Although some nursing staff naturally engage in formal and informal end-of-life communication (Phillips et al., 2020), many nursing staff are uncomfortable with end-of-life communication, lack clear guidance in clinical practice on how to engage in end-of-life communication, and are uncertain about their roles and responsibilities (Hemsley et al., 2019). To address the difficulties nursing staff experience in end-of-life communication and support them in this holistic and person-centered communication process, the fundamentals of end-of-life communication must be understood. Fundamentals include key aspects of end-of-life communication, such as the prerequisites, competencies, activities, and values involved in preparing for, carrying out, and evaluating end-of-life communication. Examples of fundamentals are, establishing a trusting relationship, moving along with the older person, creating a calm atmosphere and listening.

Previous research has identified both similarities and differences between nursing staff, older people, and family caregivers regarding how they prioritize these fundamentals. For example, while some nursing staff emphasize structured end-of-life conversations with specific outcomes, detailing the competencies, techniques, and steps they use to achieve them (Peerboom et al., 2024 Gonella et al., 2020, Punia et al., 2024), older people and their family caregivers tend to prioritize connecting and the experience of being seen and heard in ongoing, holistic, natural, and humane end-of-life conversations (Peerboom et al., 2025), where readiness for end-of-life communication is an important prerequisite (Pun et al., 2023, Gao et al., 2024, Hutchison et al., 2017, Rezaei et al., 2025;Peerboom et al., 2025). Older people and family caregivers also seem to have few expectations and little to no preparation for end-of-life conversations and approach them with an open mind (Peerboom et al., 2025) (Appendix A).

Understanding the fundamentals of end-of-life communication by nursing staff, such as through developing a theoretical framework, can enhance nurse education by guiding curricula and strengthen research through standardized measures. It can also support practice directly by preparing nursing staff for effective, person-centered end-of-life communication and care, and by informing the design and implementation of interventions that enable nursing staff to adopt a key role in these conversations. Therefore, by integrating the findings of three of our previous studies (Peerboom et al., 2023, Peerboom et al., 2024, Peerboom et al., 2025), we built a preliminary theoretical framework of the fundamentals of end-of-life communication including the perspectives of nursing staff, older people and family caregivers (Box 1 & Appendix A). Reflection on this framework from nursing staff, older people and family caregivers is necessary to verify that these previous findings are integrated correctly and enrich the framework.

The previous studies have also shown that nursing staff view spiritual caregivers and physicians as their most important collaborators in advance care planning, as these professionals can provide enriching perspectives on their roles and responsibilities and the fundamentals of end-of-life communication (Peerboom et al., 2023, Peerboom et al., 2024). Indeed, end-of-life communication requires an interdisciplinary and collaborative approach, and different disciplines might have different perspectives. For example, physicians typically approach end-of-life communication from a medical perspective, emphasizing structured conversations, decision-making about life-sustaining treatments, and prognostic information, often using a more formal tone than nursing staff (Kwak et al., 2021, Fulmer et al., 2018, Goswami, 2021). Spiritual caregivers generally focus on the patient’s spiritual and cultural context, exploring existential values, fears, and their relationship to a prognosis. They support patients emotionally during medical decision-making, help reassess wishes when care goals change, and employ deep listening techniques to facilitate reflection and expression (Kwak et al., 2021). The preliminary theoretical framework lacks the perspectives of these professionals. Therefore, this study aimed to enrich and provide a multiple discipline perspective on the preliminary theoretical framework for end-of-life communication by nursing staff as part of advance care planning with older people. In this study, multiple discipline refers to individuals involved in advance care planning, including professionals, older people and their family caregivers.

Box 1 **The LISTEN project and its preliminary framework.**

This study is part of the LISTEN project, which aims to build and validate a theoretical framework of the fundamentals of end-of-life communication between nursing staff and older people. Three studies predate the current study in identifying fundamentals of end-of-life communication by nursing staff, including a scoping review (Peerboom et al., 2023) and two interview studies with nursing staff (Peerboom et al., 2024) and older people and family caregivers (Peerboom et al., 2025). The findings from the three previous studies were integrated to create a preliminary theoretical framework of 5 themes (“Feeling comfortable”, “Creating space for open communication”, “Using senses and applying communication techniques”, “Following the conversational phases”, “Being aware of interprofessional collaboration”) encompassing 20 fundamentals.

The framework emphasizes that nursing staff should feel comfortable to talk about the end-of-life, in both private and professional contexts. For example, experiencing a trusting relationship and learning by doing can help nursing staff feel comfortable and engage in natural and open end-of-life conversations. In addition, nursing staff can create space for open communication by, for example, moving along with the older person and being easily approachable. When engaging in the conversation, nursing staff may use their senses and apply appropriate communication techniques, such as by listening and using their intuition. Although end-of-life communication does not need to follow strict phases, certain phases may be identified. Nursing staff prepare, build, and evaluate the conversation as part of a continuous process. Interprofessional collaboration is important in each part of the communication process. Nursing staff should be aware of their own role in end-of-life communication and work together based on these roles. In addition, family caregivers should be actively involved while ensuring that the older person remains central to the care process.

Alt-text: Unlabelled box

## 2 Methods

### 2.1 Design and method

Five semi-structured focus group interviews were conducted, each with different participants (Nyumba et al., 2018). Initially, four homogenous focus group interviews (nursing staff (1), spiritual caregivers (2), patient and family caregiver representatives (3), and physicians (4)) were conducted to allow for in-depth discussion of the preliminary framework for each discipline. After analyzing the data from these interviews and adjusting the preliminary framework according to this analysis, a heterogenous focus group session, including all disciplines, was conducted as a final reflection on the framework. We aimed to include 6 to 8 participants in each focus group. None of the participants had been involved in the LISTEN project before.

This study is reported according to the Consolidated Criteria for Reporting Qualitative Research (COREQ) guidelines (Tong et al., 2007).

### 2.2 Sample

This study included a purposive sample of nursing staff with diverse education levels, settings, and years of experience in palliative care. The inclusion criteria were as follows: 1) working as a nurse assistant (care assistant or certified nursing assistant); nurse (licensed vocational nurse, registered nurse, or clinical nurse specialist); or nurse practitioner in home care, nursing home, or hospital settings and 2) involvement in end-of-life communication with older people in the past 6 to 8 weeks. Older people were 65 years or older and receiving care or palliative care in home care, nursing home, or hospital settings (e.g., due to frailty or chronic illness). Participants were eligible regardless of years of experience, allowing the sample to reflect a diverse range of expertise.

This study also included purposive samples of spiritual caregivers and physicians with varying years of working experience in palliative care, work settings, and specialties. Participants were included if they 1) worked as a spiritual caregiver, elderly care physician or resident elderly care physician (Koopmans et al., 2017), general practitioner, medical specialist, or palliative care specialist in home care, nursing home, or hospital settings and 2) had been involved in end-of-life communication in the previous 6 to 8 weeks. Participants were eligible regardless of years of experience, allowing the sample to reflect a diverse range of expertise.

A convenience sample of patient and family caregiver representatives was included. Participants varied in age, gender, education level, and healthcare setting. Patient and family caregiver representatives were included if they had experience with palliative care or end-of-life communication. The heterogeneous focus group only included patient representatives with experience with focus group interviews (e.g., in advisory boards of research projects or healthcare organizations), as these interviews could be overwhelming otherwise. In the homogenous focus group interview, this was not applied as an exclusion criterium.

### 2.3 Working group

The research group consulted an interprofessional working group (n = 16) contributing to the overall LISTEN project. This group comprised a patient representative; nursing staff members with different educational levels working in hospital, home care, and nursing home settings; members of a transmural palliative care consultation team; a spiritual caregiver; and other experts in palliative care, geriatric nursing care, and nursing education. The working group was consulted twice to discuss the content of the interview guide, participant selection, the analysis approach, and the interpretation of the results of the focus group interviews.

### 2.4 Recruitment

The research team and working group recruited participants from their professional networks, professional network organizations, and palliative care network organizations. FP approached potential participants by email, started and completed the informed consent procedure, and scheduled focus group interviews with those who were interested.

### 2.5 Preparation and interview guide

Participants were required to review written information about the preliminary framework, which they received at least 1 week before the focus group interview (Appendix A). This information helped participants approach the fundamentals and framework from their personal and professional viewpoints, fostering meaningful discussion during the focus group interviews. The information included the framework’s overarching themes, fundamentals, and participant quotes from our previous interview studies (Peerboom et al., 2024, Peerboom et al., 2025). Participants in the homogenous focus group interviews also received information about the perspectives of nursing staff, older people and family caregivers from the LISTEN project’s earlier studies (Appendix A.1). Participants in the final heterogenous focus group interview received information about the changes made after the homogenous focus group interviews and an adapted version of the preliminary framework (Appendix A.2).

To guide the focus group interviews and stimulate discussion, the research group developed an interview guide (adjusted to the participants’ disciplines) in co-creation with the working group (Appendix B). The interview guide included questions on the comprehensiveness, understandability, and applicability of the preliminary framework and its fundamentals; perspectives of nursing staff, older people and family caregivers from our previous studies (only in the homogenous focus groups); interprofessional collaboration; and what nursing staff can learn from other disciplines in end-of-life communication and vice versa. Several examples are as follows: “In terms of the framework as a whole, what are your thoughts?”, “Are there any fundamentals you feel are missing when viewing the framework from the perspective of your discipline?”, and “What could you learn from nursing staff in end-of-life communication? And what can nursing staff learn from you?”. The interview guide was not pilot tested, but it was reviewed and adjusted as necessary after each focus group interview (e.g., closer examination of participants’ opinions on a specific fundamental).

### 2.6 Data collection

Two moderators conducted the focus group interviews; at least one moderator was a qualitative researcher from the research team (FP, a female nurse scientist and Ph.D. candidate). The other moderator was either another qualitative researcher from the research team or someone from the same discipline as the participants in the focus group interview.

Before the interviews, the moderator asked participants to sign the informed consent form and complete a short questionnaire about their baseline characteristics (i.e., gender, age, and healthcare setting). In addition, a moderator asked whether the preparatory information about the framework was clear or needed additional explanation. The heterogenous focus group interview began with a brief presentation explaining the reasons for adjustments to the preliminary framework based on the homogenous focus group interviews. The focus group interviews were audio-recorded and transcribed verbatim.

### 2.7 Data analysis

Data were analyzed using a framework analysis method (Goldsmith, 2021, Gale et al., 2013). Framework analysis is a comparative form of thematic analysis that uses an organized structure of inductively and deductively derived themes to conduct cross-sectional analysis combining data description and abstraction (Goldsmith, 2021). This method allows key patterns to be identified, described, and interpreted within and across disciplines and fundamentals (Goldsmith, 2021). The steps of this approach are displayed in Table 1. Atlas.ti (version 25.0.1) was used to support the analysis.

**Table 1:** Stages in the framework analysis.

**Stages**	**Approach**
1. Transcription	Each focus group interview was transcribed verbatim by an independent professional transcription service immediately after the interview. Transcripts were not returned to the participants.
2. Familiarization	FP listened to each audio recording and read the transcript at least once before proceeding to Stage 3. Analytical notes, thoughts, ideas, and impressions were recorded in memos at every stage. FP regularly reflected on these memos for rigor and reflexivity purposes.
3. Coding and developing a coding framework	FP read the transcripts line by line and independently applied open coding and deductive coding to the first four transcripts in Atlas.ti (homogenous focus group interviews). JF read two transcripts line by line and independently applied open coding and deductive coding in Atlas.ti. Deductive coding was based on the codes and fundamentals defined in the preliminary theoretical framework (coding framework, Appendix C). After analyzing two transcripts, FP and JF compared the codes they used in their analysis and agreed on a set of codes that were added to the coding framework. The additional codes were grouped into new fundamentals or added to existing fundamentals. After FP analyzed the third and fourth transcripts, the research group agreed on a set of codes, which were added to the coding framework. Again, the additional codes were grouped into new fundamentals or added to existing fundamentals. The fundamentals were then clearly defined. This formed the final coding framework.
4. Applying the coding framework	FP used the final coding framework to adjust the preliminary theoretical framework where necessary. This adapted theoretical framework was used as the basis for the final (heterogenous) focus group interview (Appendix A.2). The final coding framework was also applied to analyze the transcript of this final focus group interview. Inductive coding was applied to find possible additional codes and fundamentals.
5. Charting data into the framework matrix	FP used a spreadsheet to create a matrix in which the data were charted. Charting involved summarizing data by fundamentals from each transcript, balancing data reduction with preserving the original meaning and ‘feel’ of the interviewees’ words. The chart included references to illustrative quotes.
6. Interpreting the data	The research group identified characteristics of and differences between data, and mapped connections between fundamentals to explore relationships.

### 2.8 Rigor and trustworthiness

We ensured rigor by applying established trustworthiness criteria (Korstjens and Moser, 2018). Credibility was aimed for by conducting multiple focus groups with diverse participants, probing by the interviewers, and investigator triangulation in coding and theme development. Transferability was supported by providing rich descriptions of the study context, participants, and procedures. Dependability was addressed through documenting key methodological decisions. Confirmability was enhanced through reflective memos and peer debriefing, ensuring that findings were grounded in participants’ accounts. Ongoing reflexivity helped us critically examine assumptions and positionality, thereby strengthening transparency and rigor.

### 2.9 Ethical issues

This study followed the principles of the Declaration of Helsinki and the Medical Research Involving Human Subjects Act (WMA, 2013). It was approved by the Research Ethics Committee of the Faculty of Health, Medicine, and Life Sciences (FHML-REC) of Maastricht University (FHML-REC/2024/072).

## 3 Results

### 3.1 Participants and demographic data

Five focus group interviews were conducted between October and December 2024. The mean duration was 80 minutes. Four focus group interviews were conducted in person in a healthcare facility meeting room. One focus group interview, with physicians, was conducted online because of scheduling difficulties. In total, 10 nursing staff members, 8 physicians, 7 spiritual caregivers, and 8 patient-family caregiver representatives participated (Table 2). The participating healthcare professionals were employed at five different healthcare organizations. Two physicians declined to participate—one without explanation and the other for personal reasons. Two nurses and six physicians did not respond to our invitation.

**Table 2:** Demographic data.

	**Focus group 1** (n = 8)	**Focus group 2** (n = 6)	**Focus group 3** (n = 5)	**Focus group 4** (n = 5)	**Focus group 5** (n = 9)
**Discipline** (n)	Nurse (6) Nurse assistant (1) Nurse practitioner (1)	Patient representative (6)	Spiritual caregiver (5)	General practitioner (1) Resident elderly care physician (4)	Nurse (1) Nurse assistant (1) Patient representative (1) Family caregiver representative (1) Spiritual caregiver (2) Elderly care physician (1) Internist (1) Pulmonologist (1)
**Age** (median years (range))	37.5 (23–59)	73 (67–81)	57 (47–63)	32 (26–64)	52 (35–70)
**Sex** (n)					
Male	0	2	1	2	2
Female	8	4	4	3	7
**Setting** (n)					
Hospital	3	N/A	2	1	3*
Nursing home	3	N/A	2	3	3*
Home care	2	N/A	1	1	1*
**Experience with palliative care** (median years (range))	17 (2–40)	N/A	2 (1–23)	4 (2–33)	10 (5–30)*

*Based on the participating healthcare professionals

### 3.2 Findings

The findings from the focus group interviews are organized by theme and fundamental within the analytical and theoretical framework. Table 3 provides an overview of these findings and the changes made to the framework. The first and the last columns encompass the revised theoretical framework. Below, we highlight the results showcasing enriching perspectives from the participants regarding the following themes: “Feeling comfortable”, “Creating space for open communication”, “Using senses and applying communication techniques”, “Following the conversational phases”, “Being aware of interprofessional collaboration.”

**Table 3:** Summary of findings and revised theoretical framework.

Theme	Preliminary fundamental^1^	Summary findings homogenous focus group interviews^2^^,^^3^	Summary findings heterogenous focus group interview^2^	Revised fundamental^4^
**Feeling comfortable**	Being able to talk about the EOL	EOL communication requires awareness and personal and personal reflection (NS, PR, SC, PH)	All participants agreed with the content and added value of this fundamental.	**Self-efficacy** to talk about the EOL
		Nursing staff should be aware of what participation in EOL conversations means for and requires of them (SC).		
		**It is vital to distinguish between feeling and being able to talk about EOL** (SC)**.**		
	Establishing a trusting relationship	A trusting relationship extends beyond the basic relationship needed for a regular conversation about illness. It is built by nursing staff who show genuine curiosity about the older person and occasionally shares something personal. First impressions, shaped by body language, also play a key role (NS, PR, SC, PH).	**The sense of trust experienced in EOL communication must be felt by all involved.**	**Experiencing a mutual sense of trust**
		Nursing staff should show in EOL discussions, as this shows humanity (NS, PR, SC, PH).		
		**A trusting relationship does not necessarily need to be established but rather arises from a connection or a sense of trust in that moment** (PR, SC)**.**		
		Some older people and family caregivers find it easier to express themselves to someone with whom they do not yet have a trusting relationship (PR).		
	Learning by doing	Recognizing that ACP topics can be addressed gradually, that EOL conversations need not be difficult or controversial, and that these discussions focus on getting to know the person helps build comfort through experience (NS).	All participants agreed with the content and added value of this fundamental.	Learning by doing
		Awareness of the importance of entering a conversation in a flexible, open manner is key, regardless of the purpose of the conversation (NS).		
		The use of communication tools can be functional, but nursing staff should also be able to let go of these tools in a conversation (NS, PR, SC, PH).		
	Having a natural and open conversation	Older people and their family caregivers should feel comfortable and safe in an EOL conversation. The conversation should feel natural and emerge naturally, rather than the conversation being considered ‘good’ (NS, PR, SC, PH).	All participants agreed with the content and added value of this fundamental.	Having a natural and open conversation
		Nursing staff should be able to put older people and family caregivers at ease (SC).		
**Creating space for open communication**	Moving along with the older person	Moving along with the older person begins before a conversation starts (SC).	**Nursing staff should move along with all people involved.** Speaking clearly and tailoring communication to the person(s) involved is an important part of this fundamental.	**Attuning to** the **other person(s)**
		It is important to let go of control, avoid a fixed goal, and approach an EOL conversation as openly as possible (NS, PR, SC, PH).		
		**Alignment and awareness of cultures, norms, and values are needed** (SC, PH)**.**		
	Being easily approachable	Showing empathy should be emphasized more within this fundamental (PR).	All participants agreed with the content and added value of this fundamental.	Being easily approachable
	Creating a calm atmosphere	All participants agreed with the content and added value of this fundamental.	All participants agreed with the content and added value of this fundamental.	Creating a calm atmosphere
	Taking a seat	All participants agreed with the content and added value of this fundamental.	**An important goal of taking a seat is to create tranquility and equality.** Both contribute to seeking connection with the other person(s) in the conversation.	Taking a seat **to seek connection**
	Being honest	All participants agreed with the content and added value of this fundamental.	All participants agreed with the content and added value of this fundamental.	Being honest
**Using senses and applying communication techniques**	Listening	**Being truly, consciously present in a conversation using all senses is key to attentive listening** (NS, PR, SC, PH)**.**	All participants agreed with the content and added value of this fundamental.	**Attentive** listening
		When listening in layers, a healthcare professional may ask prompting questions to reach the existential layer of an older person’s meaning (SC).		
	Seeing	**It is important to use all the senses when entering the room where a conversation is taking place or may take place** (NS)**.**	All participants agreed with the content and added value of this fundamental.	**Non-verbal communication and observation**
	Speaking	**Silence is sometimes considered even more important than speaking in a certain way and should be added as an individual fundamental** (NS, PR, SC, PH)**.**	All participants agreed with adding “Being silent” as an individual fundamental. **Participants considered “Speaking” an important part of attuning to the other person instead of an individual fundamental.** Therefore, this fundamental was merged with “Attuning to the other person(s)”.	**Being silent**
		Silence can make older people feel seen and heard (NS, SC, PH).		
	Intuition	All participants agreed with the content and added value of this fundamental.	All participants agreed with the content and added value of this fundamental.	**Trusting** intuition
**Following the conversational phases**	Preparing the conversation	It is important to have some understanding of the person in an EOL conversation (NS, PR, SC).	All participants agreed with the content and added value of this fundamental.	**Seeking balance between preparation and flexibility**
		Preparing an EOL conversation can make the conversation more personal (PR, PH).		
		It is not necessary to prepare much for a conversation (NS, SC).		
		**If nursing staff prepare the conversation, it is crucial that they can let go of this preparation during the conversation if necessary** (PR)**.**		
		By contrast, according to physicians, it may be important to be well-informed about the details of the older person's condition and/or health (PH).		
		**Older people and their family caregivers are generally not expected to prepare for an EOL conversation** (NS, PR, SC)**.**		
		Older people and family caregivers should sometimes be prepared for a conversation about a difficult topic (e.g., CPR) by introducing it earlier (PR).		
		It is important to be aware of the general preferences and possible expectations of older people and family caregivers for EOL communication (SC).		
	Initiating the conversation	All participants agreed with the content and added value of this fundamental.	All participants agreed with the content and added value of this fundamental.	Initiating the conversation
	Gently building up the conversation	It can be easier reach the heart of the conversation in informal conversations (NS).	All participants agreed with the content and added value of this fundamental.	Gently building up the conversation
	Evaluating and following up on the conversation	All participants agreed with the content and added value of this fundamental.	All participants agreed with the content and added value of this fundamental.	Evaluating and following up on the conversation
**Being aware of interprofessional collaboration**	Perceiving your own professional role	To feel comfortable initiating an EOL conversation, nursing staff should be aware of their role in ACP, whether formally or informally (NS, SC).	All participants agreed with the content and added value of this fundamental.	Perceiving your own professional role
		Nursing staff should be allowed to talk to their patients about anything if they feel competent and confident while setting boundaries when necessary (NS, PR).		
		Nursing staff have different and often better and deeper relationships with older people than other healthcare professionals. Nursing staff help contextualize older people’s decisions and support their decision-making process (PH).		
	Involving colleagues	**Good cooperation, coordination and communication transfer between different professionals and settings is important** (NS, PR, SC, PH)**.**	All participants agreed with the content and added value of this fundamental.	**Interprofessional collaboration**
		It can sometimes be beneficial to have EOL conversations together with another healthcare professional or to prepare the conversation together (SC, PH).		
		Nursing staff should help colleagues and trainees recognize their role in ACP by sharing their experiences and providing support in EOL conversations (NS).		
		It is important to be aware of the emotional and mental impact that EOL conversations can have on oneself and colleagues (NS).		
	Involving family caregivers	All participants agreed with the content and added value of this fundamental.	All participants agreed with the content and added value of this fundamental.	Involving family caregivers

NS, nursing staff

PR, patient and family caregiver representatives

SC, spiritual caregivers

PH, physicians

EOL, end of life

ACP, advance care planning

1 Preliminary fundamentals are those originally defined in the preliminary framework and later discussed in the focus group interviews, where their title and/or content could be revised.

2 Bold text refers to the most important reasons for adapting the fundamental.

3 Abbreviations following the findings indicate the discipline that reported this in the homogenous focus groups.

4 Bold text refers to the changes made in the fundamental.

#### 3.2.1 Overall remarks on the framework

The participants in the homogeneous focus group interviews were very satisfied with the preliminary theoretical framework. They found it clear, convenient, well-organized, and recognizable. They mentioned that the framework applies to any setting and nursing staff with any educational level. Heterogeneous focus group participants reflected on the framework from a more overarching perspective. They viewed the fundamentals within the framework as building blocks that raise awareness of what is important in end-of-life conversations, specifically mentioning that not all fundamentals must be present in every end-of-life conversation; rather, they should be tailored to the conversation’s setting and the people involved. They also noted that the framework addresses both formal and informal end-of-life communication but does not explicitly differentiate between these two, causing confusion about its goal of addressing both forms. In addition, they observed that the fundamentals vary in terms of levels (i.e., some represent prerequisites and others relate to more specific actions in end-of-life communication) and that some of the titles of the fundamentals did not fully capture their scope.

#### 3.2.2 Theme 1: Feeling comfortable

##### 3.2.2.1 *Self-efficacy to talk about the* end of life

To engage in end-of-life communication, the participants in all five focus groups mentioned that nursing staff must have previously reflected on the general importance of end-of-life conversations and their own personal values, needs, wishes, or existential questions. This requires awareness and professional and personal reflection.

*“If you have never thought about it yourself, how are you going to discuss it with the client?”* (nursing staff)

Spiritual caregivers mentioned that it is important to distinguish between feeling able and being able to talk about the end of life. According to spiritual caregivers, ‘being able’ refers to skills and competencies, whereas ‘feeling able’ refers to how a nursing staff member feels when end-of-life communication occurs. Nursing staff should reflect on this feeling before the conversation.

“*Being able has to do with skills and expertise. Feeling [able] has to do with me. There are days I do not go to certain patients [because it does not feel right].*” (spiritual caregiver)

##### 3.2.2.2 Experiencing a mutual sense of trust

The focus groups of patient representatives and spiritual caregivers discussed how realistic it is in practice to establish a trusting relationship, especially with the decreasing continuity among nursing staff across healthcare settings. Both groups of participants believed that this relationship does not necessarily have to be established; rather, an end-of-life conversation arises from a sense of trust in that moment. The participants in the heterogeneous focus group added that everyone involved in the conversation must feel this trust. In addition, some patient representatives stated that it was easier to express themselves to someone with whom they did not have a trusting relationship, as this felt less intimidating.

“I *think it also has to do with the click you have with someone. […] Yes, a bond of trust can develop. But it does not have to be very long-term. It does not have to be called a relationship. Then I think it is too heavy. But sometimes it happens. Then you meet someone and they say something. You respond to that. And a conversation develops because of the sense of trust*.” (patient representative)

To establish a sense of trust, spiritual caregivers indicated that it is important for nursing staff to have the courage to reveal their vulnerability. They referred to this as professional intimacy or professional vulnerability.

“*[It is important] that as a nurse you dare to be vulnerable. Vulnerability evokes vulnerability. And if you can handle that professionally, then you can build bridges with people. I think that is very important. Not from a distance but looking for closeness. You look for the intimacy, the professional intimacy. Because that is where the conversation takes place.*” (spiritual caregiver)

##### 3.2.2.3 Learning by doing

Nursing staff explained that awareness regarding the following helped them build comfort: advance care planning topics do not need to be covered all at once but may take more than one conversation; end-of-life conversations do not have to be difficult or controversial; and end-of-life communication is primarily about getting to know someone. They learned this by doing.

“*It should not be so charged. If you put such a stamp on the conversation, then you are already starting with certain feelings. [...] The patient feels that.*” (nursing staff)

##### 3.2.2.4 Having a natural and open conversation

All participants agreed that it is crucial for older people and their family caregivers to feel comfortable and safe in an end-of-life conversation; the conversation must feel natural and emerge organically, rather than aiming to have a “good” conversation.

“*I think especially when a patient, a resident, feels completely comfortable and can be open about what he wants, then I have a good conversation. And I think certainly in the conversations that happen spontaneously, a resident often feels more comfortable sharing something*.” (physician)

#### 3.2.3 Theme 2: Creating space for open communication

##### 3.2.3.1 Attuning to the other person(s)

All participants agreed on the importance of letting go of control and a fixed goal in the conversation. Spiritual caregivers added that, unlike many nursing staff, they often have no explicit goal or intended outcome in end-of-life conversations beyond seeking connection. Nursing staff could learn from this approach.

“*[It is important] not to aim at something, but really giving space here and now to what arises—from the search for connection.*” (spiritual caregiver)

Spiritual caregivers and physicians mentioned that alignment and awareness of cultures, norms, and values are also needed in end-of-life communication. Nursing staff should be aware of their culture, norms, and values, as well as those of the older person and how they relate to each other. It is essential to recognize how differences may influence the conversation and adapt the conversation accordingly.

"*Cultures also require attunement—attunement and awareness—that death, for example, can be taboo for many people. We have to be aware of that. [...] But we should not fill in. [...] Then you have to go back and slow down. And attune. That is where it starts—your own open attitude.*” (spiritual caregiver)

Heterogeneous focus group participants added that nursing staff should not only attune to the older person, but also to family caregivers and others involved in end-of-life communication. Speaking in an understandable way, tailored to these persons involved, was also considered important in this fundamental.

“*I can imagine you have conversations with loved ones as well. And you want to move with them as well. So maybe it is better to move with the person. Otherwise, you immediately define someone as an older person. And that person is still just a person*.” (spiritual caregiver)

##### 3.2.3.2 Being easily approachable

Patient representatives indicated that showing empathy was something that needed to be emphasized more within this fundamental.

“*I missed one word in the whole piece [framework], and that is empathy. Someone who is in healthcare must carry a huge load of empathy. Otherwise, they are not going to get there. And I often missed that in [nursing staff at] the hospital. But even here [in the framework], I miss it. Maybe they have become different words. Maybe they mean the same thing. For me, empathy is the word. [...] Then you get a little bit further. A businesslike conversation does not work*.” (patient representative)

#### 3.2.4 Theme 3: Using senses and applying communication techniques

##### 3.2.4.1 Attentive listening

All participants emphasized the importance of attentive and deep listening and making the older person feel that they are being heard. Even more important was being truly, consciously present in a conversation using all your senses.

“*I always say, [...] ‘stop, look, go’ before you go in. Wait a minute. Enter the resident’s, patient’s, client’s room with all your attention. And I think that is something you have to teach yourself, to actually be there, in that moment.*” (nursing staff)

“*To really be one hundred percent with the other person and to resonate with that. What is happening there, what is being experienced there, having a kind of curiosity about that as well. Wanting to be there.*” (spiritual caregiver)

Spiritual caregivers also explained how they listen in layers, asking many prompting questions to reach the existential layer of what the older person is trying to say.

“*For me, deep listening is....listening to everything else that is going on in the conversation. So, well, partly it is listening in layers. Let me just say that you go from facts—feelings—to this existential layer. So, you look at what is underneath? And that you are almost trained to get there pretty quickly, if possible*.” (spiritual caregiver)

##### 3.2.4.2 Non-verbal communication and observation

Nursing staff expressed the importance of using all their senses when entering the room where the conversation will or may take place. They focus not only on the nonverbal expressions of the older person but also on details in the room, such as paintings or pictures.

“*You actually do a lot of other things as well. [...] Also, look around a room. What do you see in pictures? Or paintings? Or a chair? It can tell you so much. It is just really important that when you walk into a room, you really look around. Go in with all your senses*.” (nursing staff)

“*Before I enter a room and I am still standing in front of the door, I take several deep breaths to ground myself. […] Then I very consciously grab the handle to enter the room. And before I introduce myself or whatever, [...] I look around. Who is sitting there? How are they sitting?*” (spiritual caregiver)

##### 3.2.4.3 Being silent

All participants felt that at times, silence was even more important than speaking, as it could make older people and their family caregivers feel seen and heard by nursing staff, spiritual caregivers, and physicians. Spiritual caregivers viewed silence as expanding the other person’s space by reducing their own.

“*You are trying to expand the other person's space. You only do that by reducing your own space, and that means not talking so much or asking questions to teach the other person to explore their own space.*” (spiritual caregiver)

#### 3.2.5 Theme 4: Following the conversational phases

##### 3.2.5.1 Seeking balance between preparation and flexibility

Typically, nursing staff and spiritual caregivers did not feel the need to prepare much for a conversation, as this might result in a less open and natural conversation. Patient representatives added that if nursing staff want to prepare the conversation, they must let go of their agenda during the conversation if necessary.

“*Yes [nursing staff should prepare for an end-of-life conversation]. But then he or she must have the flexibility to deviate from it.*” (patient representative)

Physicians were surprised that nursing staff in our previous studies indicated that they could not (and would not) prepare thoroughly for end-of-life conversations because they felt dependent on the older person for the content of the conversation. By contrast, physicians stated that they wanted to be well-informed about the details of the older person's condition and health, such as the course of their illness, previously discussed wishes, and topics to be discussed. The physicians proposed that this contrast is mainly due to their different goals in end-of-life communication (i.e., focusing on decision-making from a medical perspective rather than a holistic discussion of needs and preferences).

Nursing staff, physicians, and spiritual caregivers did not expect older people and their family caregivers to prepare for end-of-life conversations. This could make the conversation feel unnatural and burdensome, making it harder to reach its purpose.

“*I think, ‘How can you [the older person] prepare for it?’ It should not be forced because then you do not get to the heart of what you want to talk about. [...] If somebody sits there really prepared, it is just reading off a list of what they might have written down with their children, not getting to the heart of it.*” (nursing staff)

##### 3.2.5.2 Gently building up the conversation

Nursing staff reported that informal conversations help them reach the heart of the conversation and can increase older person's readiness to talk about the end of life.

“*I think a lot of times with the informal conversations....that you can get to the heart much more often, much deeper, than the formal scheduled conversations. [...] Then, you are also more often in a much more intimate atmosphere, for example, when showering someone or whatever*.” (nursing staff)

#### 3.2.6 Theme 5: Being aware of interprofessional collaboration

##### 3.2.6.1 Perceiving your own professional role

Although nursing staff found it difficult to define their role, they felt it was more important to recognize that they have a role in advance care planning, which is not always acknowledged among their peers. This awareness would make them feel more comfortable initiating end-of-life conversations, especially informal ones. Nursing staff and patient representatives questioned whether it was necessary or realistic to delineate this role, mainly because nursing staff play different roles between and within settings, which often involve slightly different conversations. Nursing staff and patient representatives felt that nursing staff should be free to talk to their patients about anything, provided they feel competent and confident and set boundaries when necessary. Similarly, they should avoid discussing topics on which they do not have adequate knowledge.

“*I also think it is very important that when you talk about something, you have the knowledge and expertise about it. […] You [nurse] have to know what you are talking about.*” (nursing staff)

“*You have to be strong and confident enough to do it yourself. And then, yes, then where is the limit? That also has to do with self-assessment*.” (patient representative)

The focus group interview with physicians underscored that they were very aware of their role in advance care planning. They felt responsible for identifying and recording the older person's treatment preferences and ceiling of care. Some physicians indicated that they were not aware that nursing staff play a role in end-of-life communication. Speculation particularly focused on the deeper and often stronger relationships that nursing staff have with older people compared with physicians. Physicians indicated that nursing staff help contextualize older people’s stories, clarify their preferences and decisions, and provide a holistic view.

“*Every situation ... it is a story. The same problem can be discussed in endless ways. You can just describe the symptoms in a very superficial way, or you can really show in the way you talk about it that you know the person. And you can also add some elements that the patient may have shared with the nurse or care assistant during a conversation. That adds color to the symptoms and the situation.*” (physician)

##### 3.2.6.2 Interprofessional collaboration

All participating professionals emphasized that advance care planning is a team effort involving all care providers for older people not just direct colleagues. Physicians and spiritual caregivers indicated that involving nursing staff in end-of-life conversations or preparing the conversation with them can be helpful. Nursing staff often know older people and their family caregivers better than the physician or spiritual caregiver—they have probably discussed the end of life with the older person before, and they can keep the conversation light-hearted and follow up informally.

“*I always like it when nursing staff are present during my [*end-of-life*] conversations [with older people] because I notice that nursing staff often know the residents a little better. And somehow, such a conversation is a little more light-hearted. [...] It is easier for them to walk by a resident an hour later and say, ‘How are you doing now? How did you experience it [the* end-of-life *conversation]?'*” (physician)

Nursing staff also felt that it was important to make colleagues and nursing staff trainees aware of their role in end-of-life communication and support them by sharing their experiences of these conversations. In addition, they considered it important to be aware of the emotional and mental impact that end-of-life conversations have on themselves and their colleagues.

“*Take them [fellow nursing staff] to an [end-of-life] conversation, but then discuss it very carefully afterward. ‘What happened? And what happened to you?’ [...] And also bring nursing students and make them feel that the most important thing is just to be there with real attention. If you forget to ask or say something, you can always do that but just be there.*” (nursing staff)

## 4 Discussion

This study provided a multiple discipline perspective on a preliminary theoretical framework for end-of-life communication by nursing staff as part of advance care planning with older people. It confirms our findings from previous studies (Peerboom et al., 2023, Peerboom et al., 2024, Peerboom et al., 2025), which explored the perspectives of nursing staff, older people, and their family caregivers through individual interviews and a review of the scientific literature. The findings emphasize the importance of having a natural and open conversation, attuning to the other person, listening attentively, and collaborating with other professionals. However, this study also enriched our framework and understanding of end-of-life communication by nursing staff from a multiple disciplines’ perspective. For example, we found that personally *feeling* able to talk about the end-of-life and consciously reflecting on this feeling from a nursing staff perspective are at least as important as the ability to discuss end of life per se, as also supported by other studies (Ten Koppel et al., 2019, Bandura and Wessels, 1994, Kim et al., 2023). Moreover, our findings add to those of previous studies examining older people’s readiness to talk about the end of life (Rezaei et al., 2025, Gao et al., 2024), although these studies often overlook nursing staff’s readiness for these conversations. By reflecting on feeling able to talk about the end of life and acting accordingly, nursing staff may alleviate the psychological burden of end-of-life communication (Souza et al., 2024) and improve mutual trust between nursing staff, older people, and their family caregivers in these conversations.

This study emphasized the importance of experiencing mutual trust, a factor less highlighted in other studies. Although many studies (Goswami, 2021, Kwak et al., 2022), including ours (Peerboom et al., 2023, Peerboom et al., 2024, Peerboom et al., 2025), recognize the importance of patient–provider relationships in end-of-life communication, this study found that the mutual trust experienced at the inception of an end-of-life conversation was deemed at least as important as the process of building a trusting relationship over time. Participants believed that an end-of-life conversation arises from a sense of trust in that specific moment. Without this trust, a “deep” end-of-life conversation is unlikely to emerge. Some patient representatives even mentioned they sometimes find it easier to express themselves to someone with whom they do not have a trusting relationship, as this may feel less intimidating. These insights make adhering to this fundamental more realistic; for example, nursing staff often do not have enough time to build a long-term relationship with older people in hospital settings, and the lack of continuity in healthcare makes establishing such relationships challenging. Our findings also emphasize that any nursing staff member, in any setting, can have an end-of-life conversation with an older person when mutual trust exists in the moment. This highlights the importance of nursing staff being open to and aware of cues from older people, and willing to initiate informal conversations to promote end-of-life communication in clinical practice. However, this fundamental may also be person-specific; for some older people, family caregivers, and nursing staff, establishing a trusting relationship over time may still be an important facilitator of end-of-life conversations, as also emphasized by other studies (Stenman et al., 2023, Gonella et al., 2020). Nursing staff may use their senses to determine this and adjust their approach accordingly.

Participants in this study also emphasized the importance of nursing staff engaging in informal end-of-life conversations. Such conversations feel more laid-back and approachable than formal ones and may help prepare older people for the latter. Studies on confidential conversations report similar findings, showing that patients often disclose personal and existential issues, gaining emotional support and help in processing end-of-life concerns (Stenman et al., 2023, Stenman et al., 2025). In addition, physicians and spiritual caregivers in the present study noted that, among other things, nursing staff members’ informal and lighthearted approach to end-of-life conversations made them more approachable and trustworthy to older people and their family caregivers compared with other healthcare professionals; this finding is also supported by other studies (Olshansky, 2011, Milton, 2018, Bolt et al., 2021). Spiritual caregivers emphasized that they approached end-of-life conversations by letting go of rigid goals or intended outcomes, instead prioritizing seeking connection and attuning to those involved. Through this approach, nursing staff can make their conversations with older people and their family caregivers more accessible and suitable (Ke et al., 2015). This approach also better addresses the needs of older people and their family caregivers at that moment and can make them feel seen and heard, as highlighted in our previous study (Peerboom et al., 2025). Prioritizing seeking connection and attuning to the persons involved in the conversation may require nursing staff to adopt a different attitude than they are accustomed to in end-of-life communication. Nursing staff are typically very proactive professionals, and they may need to step back, reflect, remain silent, and sit down to connect with older person and their family caregivers.

These insights align with broader differences we found between the different disciplines in this study regarding their approaches to prepare for end-of-life conversations. Nursing staff and spiritual caregivers frequently preferred minimal preparation as they were concerned that extensive planning might compromise authenticity and responsiveness in end-of-life conversations (Kwak et al., 2021;Peerboom et al., 2023, Peerboom et al., 2024. Patient representatives acknowledged the value of preparation but emphasized the importance of flexibility in responding to patients’ needs and cues. Physicians, in contrast, tended to favor thorough preparation, including detailed knowledge of the older person’s condition, previous discussions, and specific topics to be addressed. This reflects their orientation toward structured, decision-focused communication with a formal tone and emphasis on medical goals and prognostic clarity (Fulmer et al., 2018, Goswami, 2021). Together, these perspectives illustrate a continuum: from relational attunement, as emphasized by spiritual caregivers and nursing staff, to structured medical decision-making, as prioritized by physicians, with patient representatives calling for a balance between the two. Recognizing and integrating these complementary orientations is essential for advancing nursing education and for fostering collaborative, person-centered communication end-of-life communication.

The findings of this study resulted in a new fundamental being added to the preliminary framework: ‘Being silent’. Participants emphasized that silence is even more important than speaking in a certain way. Silence helps older people and their family caregivers feel seen and heard, which aligns with findings from other studies on end-of-life communication in general (Somes et al., 2018, Gutiérrez and Paniagua, 2024). By prioritizing silence, nursing staff can focus more on listening, observing, and attuning to those involved, fostering meaningful end-of-life conversations (Shipley, 2010, Savett, 2011, Gutiérrez and Paniagua, 2024). As Laurence Savett quoted, “*Unless we learn the importance and practice of deliberate silence, engaged listening, and restrained response, we will miss the opportunity to provide our presence and comfort to those about whom we care. ... To listen, one must be present, not distracted, thinking about something or someone else; focused and reflective, not only on what is said, but also on the feelings behind what is being said* (Savett, 2011).” Although silence can be uncomfortable for both nursing staff and older people, Gutiérrez et al. argued that it is an essential element of both verbal and nonverbal communication—those who speak may perfectly control their words, whereas those who communicate control silence (Gutiérrez and Paniagua, 2024). Silence enables nursing staff to be truly present and meet the needs of older people and their family caregivers more effectively in end-of-life communication (Shipley, 2010).

In general, participants described the preliminary theoretical framework as clear, convenient, well-organized, and recognizable. However, some participants noted that the fundamentals do not explicitly differentiate between formal and informal end-of-life communication. Nursing staff engage in both forms of end-of-life communication; the key differences between the two lie in the setting, triggers, and goals of the conversation, as well as the people involved. These distinctions are particularly evident in the different conversational phases and interprofessional collaboration. For example, nursing staff are more willing and likely to prepare for formal end-of-life conversations, whereas informal conversations often arise spontaneously. In addition, family caregivers are more easily included in pre-scheduled end-of-life conversations than those that arise when nursing staff are responding to in-the-moment cues from older people during care activities (Becqué et al., 2021;Peerboom et al., 2023). The fundamentals in the theoretical framework act as building blocks for nursing staff to be aware of, learn from, prepare for, carry out, or evaluate end-of-life communication; they should not be considered items on a checklist. Above all, these fundamentals aim to raise awareness about the most important aspects of end-of-life communication. In our opinion, this does not mean that every fundamental must be equally present in every end-of-life conversation, and therefore, we do not explicitly differentiate between formal and informal communication in the framework. Van der Steen and Van den Block also stated this in their editorial, mentioning that the numerous recommendations for ideal advance care planning might deter people from initiating complex end-of-life communication (van der Steen and Van den Block, 2025). A pragmatic solution to ensure good-enough advance care planning, such as adopting a less formal approach, may make this complex process more suitable and reduce barriers for nursing staff to engage (van der Steen and Van den Block, 2025). Using the fundamentals within the revised theoretical framework, nursing staff can adopt a person-centered approach that reflects the values, needs, and preferences of the older person, their family caregiver, and themselves, as well as the specific context and environment in which the conversation occurs.

## 5 Strengths and limitations

This study has several strengths. First, this study is part of an overall project and was built upon our previous scoping review and interview studies involving nursing staff, older people, and family caregivers. Second, we included a multidisciplinary sample recruited from three healthcare settings and five healthcare organizations; this resulted in rich data from specific experiences and various professional perspectives and settings. Nonetheless, this study also has several limitations. First, because participants reflected on a previously developed framework, discussions were naturally focused on the framework’s contents. While this aligns with the study’s aim of evaluating the framework, it may have limited the spontaneous exploration of other issues. The interviewers, however, actively encouraged participants to share any additional thoughts on end-of-life communication, and researchers remained open to adjusting the framework based on these contributions. Ongoing discussions and reflections within the research and working groups helped ensure that insights beyond the original framework were considered. Second, to properly reflect on the framework, we purposively selected participants who had recently engaged in end-of-life communication in the Netherlands. In addition, all participants were comfortable with and aware of how they engaged in end-of-life communication. Excluding nursing staff who were less comfortable with end-of-life communication or had experience in countries outside the Netherlands may have resulted in a biased sample limiting diversity in perspectives on the fundamentals.

## 6 Future research

Further research is needed to validate the cultural sensitivity of the revised theoretical framework, especially considering the diverse experiences of nursing staff with end-of-life communication in countries outside the Netherlands. International expert opinions may be helpful in this regard. Once validated, the framework could be used in future research, such as analyses or reviews of end-of-life conversations. Second, the results should be translated into interventions that empower nursing staff to adopt a leading role in end-of-life communication and nursing education to improve these discussions in clinical practice.

## 7 Conclusions

This study emphasizes the importance of prioritizing a mutual sense of trust over establishing a long-term trusting relationship, of not having an intended outcome for the conversation other than to seek connection, and of silence being more important than speaking in a particular way in making older people and family caregivers feel seen and heard. Nursing staff may need to step back, reflect, remain silent, and sit down to connect with older people and their family caregivers. The revised theoretical framework can help nursing staff become aware of, learn from, prepare for, engage in, and evaluate person-centered end-of-life communication that respects the values, needs, and preferences of older people, their family caregivers, and themselves. Our findings may facilitate future education, research, and practice in end-of-life communication.

## Funding

This work was supported by the Netherlands Organization for Health Research and Development (ZonMw, grant number 10040022110005).

## CRediT authorship contribution statement

**Fran B.A.L. Peerboom:** Conceptualization, Methodology, Formal analysis, Investigation, Project administration, Resources, Writing – original draft, Visualization. **Jolanda H.H.M. Friesen-Storms:** Conceptualization, Methodology, Formal analysis, Writing – review & editing. **Jenny T. van der Steen:** Conceptualization, Methodology, Writing – review & editing. **Daisy J.A. Janssen:** Conceptualization, Methodology, Writing – review & editing, Supervision. **Judith M.M. Meijers:** Conceptualization, Methodology, Writing – review & editing, Supervision, Funding acquisition.

## Declaration of competing interest

The authors declare the following financial interests/personal relationships which may be considered as potential competing interests:

Judith Meijers reports financial support was provided by The Netherlands Organization for Health Research and Development (ZonMw). The other authors declare that they have no known competing financial interests or personal relationships that could have appeared to influence the work reported in this paper.

## Appendix Supplementary materials

Image, application 1

Image, application 2

Image, application 3
