# Supplementary material for: Conceptualizing end-of-life communication by nursing staff as part of advance care planning with older people: A multiple discipline focus group study
Source: Int J Nurs Stud Adv. 2025 Oct 25;9:100436. doi: 10.1016/j.ijnsa.2025.100436 (PMC12603746; doi:10.1016/j.ijnsa.2025.100436)
Supplement: Supplementary file 1 [file mmc1.docx]

Appendix A: Conceptual theoretical framework

Part 1: Initial conceptual theoretical framework (preparation for focus group 1-4)

Dear participant,

First of all, we would like to thank you for participating in one of the focus group interviews of the LISTEN project. In this focus group interview, we will reflect on the framework developed by the researchers of the important aspects (also called fundamentals) of end-of-life communication that nursing staff have with older people as part of advance care planning. Specifically, by these conversations we mean “proactive and timely informal and formal conversations between an older person, their family caregiver, and a healthcare professional about, for example, future EOL care, the transition to the EOL phase, and death and dying from a holistic perspective”.

This document presents the framework that will be discussed in the interview. The framework consists of themes that cluster the fundamentals of EOL communication. Quotations are used to explain the fundamentals. It is important that you review these before the interview.

In the meantime, if you have any questions about the interview, preparing for the interview and/or the LISTEN project, please feel free to contact one of the researchers (contact information is at the bottom of this page). Of course, there will also be time to ask questions at the beginning or during the interview.

We will see you soon!

Kind regards on behalf of the researchers,

Fran Peerboom

| **Framework from the perspectives of nursing staff** | | |
| --- | --- | --- |
| **Theme** | **Fundamental** | **Example quotations** |
| **Feeling comfortable** | Being able to talk about the EOL | “I can only have these conversations if I feel comfortable and safe. For me, that is the basis of a good conversation. You must feel comfortable and well so that you can listen to what the resident has to say. I think that is important.” (care assistant, nursing home)  “I am human too, right? I can also shed a tear if the person has really touched me emotionally. That should simply be possible as well.” (care assistant, nursing home)  “I have that kind of conversation with the client, and I have to give the client my full attention. To let him vent. I should not sit there with my own emotion.” (specialized nurse, nursing home)  “If you can handle it [talking about dying] privately, then you can handle it professionally as well. Yes, I think for a lot of people it is really about learning about dying. Learning to be comfortable with it. Learning to see that it is part of life and that you do not need to talk about death, you need to talk about life, and how do you want to fill in the remaining part of your life?” (nurse, hospital) |
|  | Establishing a trusting relationship | “The patient is also quick to share things on their own or during moments of care. These are certainly intimate moments for the patient, which makes them feel comfortable talking about it. […] Then I come back the next day to wash someone; then I come back to a family discussion. They often see the same face anyway. Also in the evening or even at night, when people feel uncomfortable or just cannot sleep or just want to talk to someone about it.” (nurse, hospital)  “The moment you feel that the conversation is going well and that the patient is opening up to you, then you can deepen the conversation, slowly guiding it towards what you want to know about the patient.” (nurse practitioner, hospital) |
|  | Learning by doing | “When I started doing the conversations myself […] I was not really comfortable yet because I was trying to force a certain issue. […] Then you are not comfortable, and a patient feels that. […] Yes, as I slowly drove that structure into the background, I started having completely different conversations and also started talking about life.” (nurse practitioner, hospital)  “I think you can still do so many courses and training, which might give you some tools, but if you do not feel things, then I do not think all that training is of any use at all.” (nurse practitioner, hospital) |
| **Creating space for open communication** | Moving along with the older person | “Every conversation is so different. Always so different. What you say to patient A, you cannot say to patient B. No, you cannot. […] You have to adapt to the person.” (specialized nurse, nursing home) |
|  | Being easily approachable | “I also show a piece of myself. That is very important. [...] That they feel, 'I know you.'” (specialized nurse, nursing home)  “I think it requires a big part of your empathy, but I think it also requires you to just be human and not just be the nurse who comes in to perform the technical procedures.” (nurse, hospital) |
|  | Creating a calm atmosphere | “I also actually go there and then I have the time and then I have the conversation. But I always try to do that here as well. Even though I am busy I get the feedback back, ‘You are always calm’…I say, ‘Well...you should know’. Yes, I seem to radiate that [calmness]. And I do think that is very important.” (specialized nurse, nursing home) |
|  | Taking a seat | “I never stand. No. [..] That really is a no-go. I have to be at eye level. […] I am equal to the client. It is very different talking if I stand above and...no, you cannot. Even if a client is on the floor, I still get down on my knees.” (specialized nurse, nursing home) |
|  | Being honest | “People sometimes say: you are not allowed to tell the patient that he is dying. [...] And usually, I come into the room, and you look at the person and they say: ‘I am dying, eh’. I say ‘yes’. You know, it is not up to me to say ‘no’ at that moment.” (nurse, hospital)  “I think it is my job to start the conversation about that. How things have been at home, what they expect, how they see themselves in a year's time. [...] Sometimes they tell me that ‘[in a year] I am going to take a nice trip around America with my family’. That is the point for me to jump in and marinate that patient in the idea that life might also be over sometime soon.” (nurse practitioner, hospital) |
| **Using senses and applying communication techniques** | Listening | “Listen… by keeping your mouth shut. Literally. And by simply listening to what the client says. And occasionally summarizing what you have heard. But listening really means listening. Sometimes you can really listen for half an hour and just let the client talk.” (specialized nurse, nursing home)  “You need to take your time and listen carefully to what the patient is telling you and not be busy with the next question in the conversation. That is what you often used to be busy with. The patient tells you a lot, but in your head, you think, ‘Oh, I have to ask this’, but then you miss the essence of what someone is telling you. [...] I think the main thing is not to be too preoccupied with the next question you are going to ask.” (nurse practitioner, hospital)  “Yes, what is important to the patient, what is on his mind […] you try to filter, I think. I do not think you really listen, but you filter what comes up in the conversation, what it does to the patient, what their non-verbal response to it is. And you are trying to address that.” (nurse practitioner, hospital) |
|  | Seeing | “When they talk about it [love for loved ones or pets], you can often see a certain sparkle in their eyes. [...] So, it is not just listening, it is also looking: what does it do to a patient when they start talking about something? Does someone get sad? Does someone get a smile on their face from what they are telling?” (nurse, hospital)  “You are looking for confirmation that yes, that is what I see, or this is what the patient is experiencing. [...] Maybe the patient does not realize that it makes them more emotional or that someone is very distressed or panicked by a certain idea. [...] You also try to hold up a mirror to them in the good sense of 'you say it, but I do not really see from you that it is actually true, is that correct?’" (nurse, hospital) |
|  | Speaking | “You can use 25 sentences for something, but then the essence is often lost. So, it is better to say it concretely with examples of what it is about and what we are talking about. In CPR, for example, I always appoint that the heart is stopped; they are going to press on it to get it back again.” (nurse practitioner, hospital)  “Sometimes it is good to just take a step back and let silence fall so that the client has space to move forward.” (nurse, home care)  “Allow silence in the conversation if they ask a question or they have been emotional. That too is allowed. Just be quiet and see what happens. Especially see what happens. And allow that silence for a moment. Or if you are having a conversation and you notice that someone is starting to think...and then allow that silence.” (nurse, home care) |
|  | Intuition | “This [sensing] is a nursing thing that I can never articulate very well, but we [nursing staff] all feel it.” (nurse practitioner, hospital)  “You cannot hang a protocol on it. It is often an intangible thing. It is a feeling.” (specialized nurse, nursing home) |
| **Following the conversational phases** | Preparing and expectations for the conversation | “The moment I am there, it happens. [...] You cannot prepare it because you do not know what the other person's needs are at that moment.” (nurse, home care) |
|  | Initiating the conversation | “Most of the time, it is really by chance. Today, for example, I was sitting with Mr. R. in the living room. He started himself: 'I do not remember it all'. Then I have an opening, and then I can engage in a conversation. Then I ask, 'How come, and what do you feel? How do you feel?' In that way, I then continue.” (care assistant, nursing home)  “Yes, or I or the physician will start the conversation with the situation of the last few days or weeks. Yes, what you see. These are the symptoms or the deterioration, the behavior... and the family then says: we recognize this, or we also see this or... And then, yes, eventually you get to a point where you say, ‘what are the expectations and where is this going to go?’.” (nurse nursing home) |
|  | Gently building up the conversation | “I try to keep it informal, especially when people find it very difficult to engage in difficult conversations. Then you try to give a certain level of trust through something light-hearted at first, and if you find that it is there and the conversation goes really well, then you just get really far and also really deep sometimes.” (nurse practitioner, hospital)  “Often, they will tell me who they are, what they are struggling with, what their family is like. From there, I can ask more questions. [...] And the other time, yes, it is actually like, well, I have been asked by the district nurse, for example, who has indicated that a conversation is needed. And then they start talking from there.” (specialized nurse, home care) |
|  | Evaluating and following up the conversation | “Often, in the room, I ask if everything has been discussed, if the patient has any questions. Whether there is anything else I can do for him. [...] And if not, relatively shortly at the end of my shift, I will walk in again later to ask, ‘Was the conversation okay? Are you okay?’.” (nurse, hospital) |
| **Being aware of interprofessional collaboration** | Perceiving your own professional role | “It is not entirely clear what I am really allowed to do according to certain guidelines and protocols. It really is a gray area, I think. So, you are a bit more reserved in what you say and what you ask. You also do not want to say things that are not up to me.” (nurse, hospital) |
|  | Involving colleagues | “I do that [evaluate] more often with colleagues. It may be that I also have doubts sometimes, and then I evaluate with colleagues, like ‘Look, I had this conversation yesterday, I did this and that. What do you think?' Or I do it with the spiritual caregiver.” (specialized nurse, nursing home) |
|  | Involving family caregivers | “I think it is nicer to have the conversation with the family so that they are also aware of what the client wants. Because I think it is important that we are all on the same page. But if the client does not want that, or does not have a social network, we can also do it alone.” (nurse, home care)  “What I personally do is try to stay out of…disagreements between families. That is something distressing. [...] Then I say, you know, [...] ‘you have to try to resolve that between yourselves’. [...] You do try to really focus on that client. I still focus on the first contact then, but I am not going to steer into the whole family dynamic.” (specialized nurse, nursing home) |
| **Framework from the perspectives of older people and their family caregivers**  We also interviewed older people and their family caregivers. They found it difficult to describe what they thought was important in an end-of-life conversation with nursing staff. In addition, they had few expectations for the conversation and did little or no preparation. The fundamentals that older people and their family caregivers did mention (between the lines) were similar to those listed in the table above. However, nursing staff are very aware of the many different fundamentals that are important in end-of-life conversations and want to maintain control of the conversation. Older people and their family caregivers consider this to be much less important. Above all, they want to feel comfortable, safe, seen and heard in an open, natural and human conversation.  “I think it is important to know something about the staff, reciprocally. [...] It makes you feel at home, yes. It is like being with your children. [...] It feels the way it should, right? The warmth. The warmth of two sides. [...] Then we are the same, the uniqueness of them and me. So, open towards each other. That you know each other well. [...] That also makes for a deeper conversation.” (91 years old, general practitioner office)  “They [nurses] must be very open. It [the EOL conversation] then feels so personal. It [the EOL conversation] feels just like home then. And I think that is very important.” (91 years old, general practitioner office)  “I think she [the nurse] empathizes very well. [...] Because she understands. [...] She understands your feelings, how you experience, you feel that she understands. And that gives me a very trusting impression.” (96 years old, nursing home)  “If she refers to an earlier conversation. [...] Then it has been real. Then it has remained in her mind, it has gone a little deeper. Not like in one ear and out the other. I think that is very important, to become close to someone. Yeah. Yeah, I think that is really important.” (91 years old, general practitioner office) | | |

Part 2: Adapted theoretical framework (preparation for focus group 5)

Dear participant,

First of all, we would like to thank you for participating in one of the focus group interviews of the LISTEN project. In this focus group interview, we will reflect on the framework developed by the researchers of the important aspects (also called fundamentals) of end-of-life communication that nursing staff have with older people as part of advance care planning. Specifically, by these conversations we mean “proactive and timely informal and formal conversations between an older person, their family caregiver, and a healthcare professional about, for example, future EOL care, the transition to the EOL phase, and death and dying from a holistic perspective”.

This document presents the framework that will be discussed in the interview. Adjustments have already been made in the framework as a result of the previous focus group interviews. These can be identified by the blue text. The framework consists of themes that cluster the fundamentals of EOL communication. Quotations are used to explain the fundamentals. It is important that you review these before the interview.

In the meantime, if you have any questions about the interview, preparing for the interview and/or the LISTEN project, please feel free to contact one of the researchers (contact information is at the bottom of this page). Of course, there will also be time to ask questions at the beginning or during the interview.

We will see you soon!

Kind regards on behalf of the researchers,

Fran Peerboom

| **Theme** | **Fundamental** | **Example citations** |
| --- | --- | --- |
| **Feeling comfortable** | Self-efficacy to talk about the EOL | “**I can only have these conversations if I feel comfortable and safe**. For me, that is the basis of a good conversation. You must feel comfortable and well so that you can listen to what the resident has to say. I think that is important.” (care assistant, nursing home)  “If you can **handle it** [talking about dying] **privately**, then you can **handle it professionally as well**. Yes, I think for a lot of people it is really about **learning about dying**. Learning to be comfortable with it. Learning to see that it is part of life and that you do not need to talk about death, you need to **talk about life**, and how do you want to fill in the remaining part of your life?” (nurse, hospital)  “I am human too, right? I can also **shed a tear** if the person has really touched me emotionally. That should simply be possible as well.” (care assistant, nursing home)  “I have that kind of conversation with the client, and I have to **give the client my** **full attention**. To let him vent. I should **not sit there with my own emotion**.” (specialized nurse, nursing home)  “You **do not expect that [to show emotion]**. […] But when it happens, it happens. **It is also human**, of course. [...] Because underneath everything they do, they remain a human being with their own personality. [...] Respect everyone as they are. I think that is very important.” (74 years old, general practitioner office)  "If you've never thought about it yourself, how are you going to discuss it with the client?" (nurse, focus group)  "**Do you dare? Can you do it? Is this the time?** Can you explain to your team that you've been with somebody for half an hour while other things have been delayed? Is there an understanding of that, you know? [...] There's a lot in there." (patient representative, focus group)  "***Being* able** has to do with **skills and expertise**. ***Feeling* [able]** **has to do with me**. There are days when I don't see certain patients [because it doesn't feel right].” (spiritual caregiver, focus group) |
|  | ~~Establishing a trusting relationship~~ Experiencing a sense of trust | “The patient is also quick to share things on their own or during moments of care. These are certainly **intimate moments** for the patient, which makes them **feel comfortable talking about it**. […] Then I come back the next day to wash someone; then I come back to a family discussion. They **often** see **the same face** anyway. Also in the evening or even at night, when people feel uncomfortable or just cannot sleep or just want to talk to someone about it.” (nurse, hospital)  “The moment you feel that the **conversation is going well** and that the **patient is opening up to you**, then you can de**epen the conversation**, slowly guiding it towards what you want to know about the patient.” (nurse practitioner, hospital)  “I think it is **important to know something about the staff**, reciprocally. [...] It makes you **feel at home**, yes. It is like being with your children. [...] It feels the way it should, right? The warmth. **The warmth of two sides**. [...] Then we are the same, the uniqueness of them and me. So, open towards each other. That you know each other well. [...] That also makes for a **deeper conversation**.” (91 years old, general practitioner office)  “I think it [**a humane conversation**] is also **easiest for her** [the nurse]; to see the person that way, in a normal conversation.” (75 years old, general practitioner office)  "I think it also has to do with **the click you have with someone**. Yes, a bond of trust can develop. But **it doesn't have to be very long-term**. It doesn't have to be called a **trusting relationship**. Then I think it's **too heavy**. A good sense of trust. But **sometimes it happens**. Then you meet someone and they say something. You respond to that. And a **conversation develops** because of the **sense of trust**. (patient representative, focus group)  "Your **communication starts when you walk in the door**. The first word you say. People have to look at your **body language** because that's seventy percent of the communication, **trust has to come from that**. If that trust is not there, forget it. So you **have to be very aware of how communication works**. And that's a lifelong learning process. It's never finished. That you realize how communication works. And how communication works with the elderly and the vulnerable". (spiritual caregiver, focus group)  "That as a nurse you **dare to be vulnerable**. **Vulnerability evokes vulnerability**. And if you can handle that professionally. Then you can build bridges with people. I think that is very important. Not from a distance **but looking for closeness**. You look for the intimacy, the **professional intimacy**. Because that's where the conversation takes place. (spiritual caregiver, focus group) |
|  | Learning by doing | “When I started doing the conversations myself […] I was **not really comfortable** yet because I was **trying to force a certain issue**. […] Then you are not comfortable, and **a patient feels that**. […] Yes, as I slowly drove that **structure into the background**, I started having completely different conversations and also started **talking about life**.” (nurse practitioner, hospital)  “I think you can still do so many courses and training, which might give you some tools, but **if you do not feel things, then I do not think all that training is of any use at all.**” (nurse practitioner, hospital)    “It should not remain on paper. Then there's no point.” (client representative, focus group) |
|  | Having a natural and open conversation | “They [nurses] must be **very open**. It [the EOL conversation] then feels so personal. It [the EOL conversation] **feels just like home then**. And I think that is very important.” (91 years old, general practitioner office)  “Keep **smiling**. [...] Then you also notice that it [the EOL conversation] feels **pretty relaxed** already.” (84 years old, hospital)  "I think especially when a patient **feels completely comfortable** and can be open about what he wants, then I have a good conversation. And I think certainly in the conversations that happen spontaneously, a resident often feels comfortable sharing something. (physician, focus group) |
| **Creating space for open communication** | Attuning to the older person | “**Every conversation is so different**. Always so different. What you say to patient A, you cannot say to patient B. No, you cannot. […] You have to **adapt to the person**.” (specialized nurse, nursing home)  "Not to aim something. But really **giving space here and now to what arises**. From the **search for connection**." (spiritual caregiver, focus group)  "**Cultures also require attunement**. **Attunement and awareness**. That death, for example, can be taboo for many people. We have to be aware of that. [...] But we should not fill in. [...] **Then you have to step back and slow down**. And attune. That's where it starts. **Your own open attitude**". (spiritual caregiver, focus group) |
|  | Being easily approachable | “I also show a **piece of myself**. That is very important. [...] That they feel, 'I know you.'” (specialized nurse, nursing home)  “I think it requires a big part of your **empathy**, but I think it also requires you to **just be human** and not just be the nurse who comes in to perform the technical procedures.” (nurse, hospital)  “I think she [the nurse] **empathizes very well**. [...] Because she understands. [...] She **understands** your feelings, how you experience, you feel that she understands. And that gives me a very **trusting impression**.” (96 years old, nursing home)  “You can **talk about anything** [**with the nurse**]. So that is pleasant. You can actually say, tell, whatever you want. [...] And she [the nurse] also gives her answers to that. That is pleasant.” (74 years old, general practitioner office)  "I **missed one word** in the whole piece [framework], and that is **empathy**. Someone who is in healthcare has to carry a huge load of empathy. Otherwise, they're not going to get it there. And I often missed that in [nursing staff at] the hospital. But even here [in the framework] I miss it. Maybe they have become different words [in the framework]. Maybe they mean the same thing. For me, empathy is the word. [...] Then you get a little bit further. **A businesslike conversation doesn't work either**. (patient representative, focus group) |
|  | Creating a calm atmosphere | “I also actually go there and then **I have the time** and then I have the conversation. But I always try to do that here as well. Even though I am busy I get the feedback back, ‘**You are always calm’**…I say, ‘Well...you should know’. Yes, I **seem to radiate that** [calmness]. And I do think that is very important.” (specialized nurse, nursing home)  “If such a person [the nurse] is **relaxed**, then I think the conversation also runs a little easier than with a tensed attitude. [...] That is also how **most things come to the table**.” (74 years old, general practitioner office) |
|  | Taking a seat to seek connection | “I **never stand**. No. [..] That really is a no-go. I have to be at **eye level**. […] I am equal to the client. It is very different talking if I stand above and...no, you cannot. **Even if a client is on the floor**, I still get down on my knees.” (specialized nurse, nursing home) |
|  | Being honest | “People sometimes say: you are not allowed to tell the patient that he is dying. [...] And usually, I come into the room, and you look at the person and they say: ‘I am dying, eh’. I say ‘yes’. You know, it is **not up to me** to say ‘no’ at that moment.” (nurse, hospital)  “I think it is **my job** to start the conversation about that. How things have been at home, what they expect, how they see themselves in a year's time. [...] Sometimes they tell me that ‘[in a year] I am going to take a nice trip around America with my family’. That is the point for me to jump in and marinate that patient in the idea that **life might also be over sometime soon**.” (nurse practitioner, hospital)  “Even if it is painful. Just **tell everything honestly** [during an EOL conversation]. Then you know where you stand. […] **That is how I prepare myself to be able to cope** [with the rest of the conversation and the future].” (91 years old, general practitioner office) |
| **Using senses and applying communcation techniques** | Listening | “You need to take your time and listen carefully to what the patient is telling you and no**t be busy with the next question in the conversation**. That is what you often used to be busy with. The patient tells you a lot, but in your head, you think, ‘Oh, I have to ask this’, but then you miss the essence of what someone is telling you. [...] I think the main thing is not to be too preoccupied with the next question you are going to ask.” (nurse practitioner, hospital)  “Yes, what is important to the patient, what is on his mind […] you try to **filter**, I think. I do not think you really listen, but you filter what comes up in the conversation, what it does to the patient, what their non-verbal response to it is. And you are **trying to address that**.” (nurse practitioner, hospital)  “Because she [the nurse] is **interested in you**, she **remembers** what you have, she remembers the little problems. Because it is really an art to know people well, to still remember what they actually have.” (96 years old, nursing home)  “If she **refers to an earlier conversation**. [...] Then it **has been real**. Then it has remained in her mind, it has gone a little deeper. Not like in one ear and out the other. I think that is very important, to become close to someone. Yeah. Yeah, I think that is really important.” (91 years old, general practitioner office)  “I **quickly notice** if someone is interested in what I say. I can tell very quickly. **Some people listen but are not present**. Others encourage me to go on and tell.” (86 years old, nursing home)  "I always say: [...] **'stop, look, go**’. Before you go in. Wait a minute. Enter the resident’s, patient’s, client’s room with all your attention. And I think that's something that you have to **teach yourself to actually be there** in that moment. (nurse, focus group)  "For me, deep listening is .... **Listening to everything else that's going on in the conversation**. So, well, partly it's listening in layers. Let me just say that you go from facts, feelings **to this existential layer**. So you look at what's underneath? And that you're almost trained to get there pretty quickly, if possible. (spiritual caregiver, focus group)  "That [**you should not] want to achieve a goal** in the conversation. But to really **be one hundred percent with the other person** and to resonate with that. To have a kind of **curiosity** about what is happening, what is being experienced. Wanting to be there. For me that is deep listening. [...] That they [older people] feel that they are being listened to and that they are being asked about what they are saying". (spiritual caregiver, focus group) |
|  | Seeing | “When they talk about it [love for loved ones or pets], you can often see a certain sparkle in their eyes. [...] So, it is not just listening, it is also looking: what does it do to a patient when they **start talking about something**? Does someone get sad? Does someone get a smile on their face from what they are telling?” (nurse, hospital)  “You are looking for **confirmation** that yes, that is what I see, or this is what the patient is experiencing. [...] Maybe the patient does not realize that it makes them more emotional or that someone is very distressed or panicked by a certain idea. [...] You also try to **hold up a mirror** to them in the good sense of 'you say it, but I do not really see from you that it is actually true, is that correct?’" (nurse, hospital)  "You actually do a lot of other things as well. [...] Also, **look around a room**. What do you see in pictures? Or paintings? Or a chair? It can tell you so much. It's just very important that when you walk into a room, you really look around. **Go in with all your senses**.'" (nurse, focus group)  "Before I enter a room and I'm still standing in front of the door, I **take several deep breaths to ground myself**. [...] Then I **very consciously grab the handle** to enter the room. And before I introduce myself or whatever, [...] I **look around**. Who is sitting there. How are they sitting?" (spiritual caregiver, focus group) |
|  | Speaking | “You can use 25 sentences for something, but then the essence is often lost. So, it is better to say it **concretely with examples** of what it is about and what we are talking about. In CPR, for example, I always appoint that the heart is stopped; they are going to press on it to get it back again.” (nurse practitioner, hospital)  “She [the nurse practitioner] wanted to **draw it** [the course of the disease] for him. So that he could **understand it better**. Because he said many times that he did not understand it. [...] **So she would explain it over and over again**.” (71 years old, hospital) |
|  | Being silent | “Listen… by **keeping your mouth shut**. Literally. And by simply listening to what the client says. And occasionally summarizing what you have heard. But listening really means listening. Sometimes you can really listen for half an hour and **just let the client talk**.” (specialized nurse, nursing home)  “Sometimes it is good to **just take a step back** and let silence fall so that the client has **space to move forward**.” (nurse, home care)  "I also think it's important **not to be afraid of silence in a conversation**. We don't always have to fill everything up. And I think silences are also really necessary to feel seen and heard. To get that little bit of attention. I think we also need to be more aware that that's possible. That that space is there. **The space to just be quiet**. (nurse, focus group)  "You are trying to **expand the other person’s space**. You do that only **by reducing your own space**, and that means not talking as much or asking questions to teach the other person to explore their own space." (spiritual caregiver, focus group)  "I once had coffee with a group of nurses. And they said, 'Well, I don't really know what to say, you know.’ And then I said. **I don't think you have to say anything**. (spiritual caregiver, focus group) |
|  | Intuition | “You **cannot hang a protocol** on it. It is often an **intangible** thing. It is a feeling.” (specialized nurse, nursing home)  “She [the nurse] **notices very quickly** if something is wrong.” (91 years old, general practitioner office)  “**Everyone [every nurse] is also different in this regard**. I cannot say like, ‘You [nurse] should do this or that.’ You [the nurse] must be able to **sense it a little bit**.” (70 years old, hospital) |
| **Following the conversational phases** | Preparing and expectations for the conversation | “The moment I am there, it happens. [...] You **cannot prepare** it because **you do not know what the other person's needs are** at that moment.” (nurse, home care)  “I **just let it [the EOL conversation] come to me** because I did not expect it to actually be that extensive.” (70 years old, hospital)  “We [older person and family caregiver] **went in blank**. I thought, ‘**We will see**, but it will not be fun,’ right? When she [nurse practitioner] says, ‘I want to talk to your wife and children,’ then you think ‘This must have a reason.’” (74 years old, hospital)  “So we know that she [the nurse] is coming on April 5th. […] Now should there be anything in that period up to April 5th, **we either remember it or we write it down**, like, ‘We should ask that anyway [during the next EOL conversation].’” (75 years old, general practitioner office)  "I think, how can you [older person] prepare for it? It **shouldn't be forced**, because **then you don't get to the heart of what you want to talk about**. [...] If somebody sits there really prepared, then it's just reading off a list of what they might have written down with their children, not getting to the heart of it." (nurse, focus group)  "Yes [a nurse has to prepare for an EOL conversation]. But then he or she must have the **flexibility to deviate from it**." (patient representative, focus group)  "If you are in the hospital for a certain treatment. Then one of the questions is, do you want to be resuscitated? And people haven't thought about that at all. [...] If they know that that's a question that's going to be asked, **then they can think about it**. Because I think you just **overwhelm** people with things like that." (patient representative, focus group) |
|  | Initiating the conversation | “Most of the time, it is **really by chance**. Today, for example, I was sitting with Mr. R. in the living room. He **started himself**: 'I do not remember it all'. Then **I have an opening**, and then I can engage in a conversation. Then I ask, 'How come, and what do you feel? How do you feel?' In that way, I then continue.” (care assistant, nursing home)  “Yes, or I or the physician will **start the conversation** with the **situation of the last few days or weeks**. Yes, what you see. These are the symptoms or the deterioration, the behavior... and the family then says: we recognize this, or we also see this or... And then, yes, eventually you get to a point where you say, ‘what are the expectations and where is this going to go?’.” (nurse nursing home) |
|  | Gently building up the conversation | “I try to **keep it informal**, especially when people find it very difficult to engage in difficult conversations. Then you try to give a certain level of trust through something **light-hearted** at first, and if you find that it is there and the conversation goes really well, then you just get really far and also really deep sometimes.” (nurse practitioner, hospital)  “Often, **they will tell** me who they are, what they are struggling with, what their family is like. **From there, I can ask more questions**. [...] And the other time, yes, it is actually like, well, I have been asked by the district nurse, for example, who has indicated that a conversation is needed. And then they start talking from there.” (specialized nurse, home care)  “**The more often we [older person and family caregiver] talk about it** [end of life, death], the more likely [habitual] it [having these conversations] becomes for you [family caregiver]. And also for myself [older person]. I think it is going to be **a little easier that way**.” (75 years old, hospital)  "I think a lot of times with the informal conversations.... that **you can get to the heart much more often**, much deeper than the formal scheduled conversations. [...] Then you are also in a much more intimate atmosphere, for example when showering someone or whatever." (nurse, focus group) |
|  | Evaluating and following up the conversation | “Often, in the room, I **ask if everything has been discussed**, if the patient has **any questions**. Whether there is anything else I can do for him. [...] And if not, relatively shortly at the end of my shift, **I will walk in again later to ask**, ‘Was the conversation okay? Are you okay?’.” (nurse, hospital)  “There are also people who hardly ever talk about it [the end of life]. For them, it [the conversation] makes an impact. They have to **think about [reflect on] it again**. **I do not have to think [reflect] at all**; I just have a conversation, and then it is okay.” (96 years old, nursing home) |
| **Being aware of interprofessional collaboration** | Perceiving your own professional role | “It is **not entirely clear what I am really allowed to do** according to certain guidelines and protocols. It really is a **gray area**, I think. So, you are a bit more **reserved** in what you say and what you ask. You also do not want to say things that are not up to me.” (nurse, hospital)  "I also think it is very important that when you talk about something, you have the knowledge and the expertise about it. [...] **You** [nurse] **have to know what you're talking about**." (nurse, focus group)  "You have to be **strong and confident enough** to do it yourself. And then, yes, where is the limit? That also has to do with **self-assessment**. (patient representative, focus group)  "Every situation... **it's a story**. The same problem can be discussed in endless ways. You can just describe the complaints in a very superficial way, or **you can really show in the way you talk about it that you know the person**. And you can also **add some elements** that the patient may have **shared with the nurse or care assistant** during a conversation. That **adds color to the symptoms and to the situation**. (physician, focus group) |
|  | Interprofessional collaboration | “I do that [**evaluate**] more often with colleagues. It may be that I also have doubts sometimes, and then I evaluate with colleagues, like ‘Look, I had this conversation yesterday, I did this and that. **What do you think?**' Or I do it with the spiritual caregiver.” (specialized nurse, nursing home)  "**Take them [fellow nursing staff] to an [EOL] conversation**. But then discuss it very carefully afterwards. What happened? And what happened to you? ' [...] And also **bring nursing students** and make them feel like, 'the most important thing is just to be there with real attention. If you forget to ask or say something, you can always do that but just be there.'" (nurse, focus group)  "I think part of it is also up to us as nurses to start **making those contacts** [with professionals in other settings]." (nurse, focus group)  "I always like it when nursing staff are present during my [EOL] conversations, because I notice that nursing staff often **know the residents a little better**. And **somehow such a conversation is a little more light-hearted**. [...] It is **easier for them to walk by a resident an hour later** and say: 'How are you doing now? How did you experience it? " (physician, focus group) |
|  | Involving family caregivers | “I think it is nicer to have the conversation **with the family** so that they are also aware of what the client wants. Because I think it is important that we are **all on the same page**. But if the client does not want that, or does not have a social network, we can also do it alone.” (nurse, home care)  “What I personally do is try to stay out of…disagreements between families. That is something distressing. [...] Then I say, you know, [...] ‘you have to try to resolve that between yourselves’. [...] You do try to really **focus on that client**. I still focus on the first contact then, but I am **not going to steer into the whole family dynamic**.” (specialized nurse, nursing home)  “Let him [the older person] tell, and then what needs to be added, I will add. […] Y**ou [the older person] tell your story; I will add to it if necessary**. I can also say something. And that is nice anyway.” (74 years old, general practitioner office)  “When she [the nurse] comes over, she [the nurse] comes for the patient [the older person], but she [the nurse] will also always come to me and ask, ‘How are you?’ They just know. […] That is also important, **how the person next to them [the older person] deals with it**.” (74 years old, general practitioner office) |
