# Supplementary material for: Conceptualizing end-of-life communication by nursing staff as part of advance care planning with older people: A multiple discipline focus group study
Source: Int J Nurs Stud Adv. 2025 Oct 25;9:100436. doi: 10.1016/j.ijnsa.2025.100436 (PMC12603746; doi:10.1016/j.ijnsa.2025.100436)
Supplement: Supplementary file 2 [file mmc2.docx]

Appendix B: Interview guides

Part 1: Nursing staff

| **Opening question** | |
| --- | --- |
| **From your expertise, when is a conversation a good conversation?** | |
| What words come to mind when you think about the role of nursing staff EOL communication? | |
| **Overarching questions regarding the framework** | |
| **Looking at the framework as a whole, what thoughts come to mind?** | |
| **Are all parts of the framework clear to you?** | |
| **Are there parts that you miss in the framework?** | What components are you missing? Could you please explain why? |
| Should these also be part of nursing staff’s EOL conversations? | Could you please explain why? |
| **To what extent do you find the framework applicable within each setting (i.e., hospital, nursing home, home)?** |  |
| **To what extent do you find the framework applicable to nursing staff of any level?** |  |
| **Points of discussion** | |
| Do you always engage in EOL conversations yourself? | Could you please explain why? |
| **How do you prepare yourself for an EOL conversation?** | Can/would you please explain why/how you do/how you deal with this? |
| **How do you prepare the person you are having this conversation with for an EOL conversation?** |  |
| How do you handle your own emotions during a conversation? |  |
| Do you experience differences between formal (pre-planned) and informal (spontaneous) communication? |  |
| **What are your thoughts about the differences we found in the perspectives of older people and their family caregivers and nursing staff?**   - **Different expectations.** - **Suggestion that for these conversations, nursing staff should occasionally let go of control and instead focus on listening, being present and moving with them.** - **Usually, no preparation by and few expectations from older people and their family caregivers combined with a lack of encouragement for this from nursing staff versus expecting a proactive role from them in ACP.** | Could you please explain why? |
| What tools do you use when having EOL conversations? |  |
| **From your professional role, how would you describe interprofessional collaboration as it relates to EOL communication? What do you consider important in this?** | To what extent does this differ for nurses, care assistants and nurse practitioners? |
| **Closing** | |
| Questions/reflection | |

Part 2: Spiritual caregivers and physicians

| **Opening question** | | |
| --- | --- | --- |
| **From your expertise, when is a conversation a good conversation?** | | |
| What words come to mind when you think about the role of nursing staff EOL communication? | | |
| **Overarching questions regarding the framework** | | |
| **Looking at the framework as a whole, what thoughts come to mind?** | | |
| **Are all parts of the framework clear to you?** | | |
| **Are there parts that you miss in the framework?** | What components are you missing? Could you please explain why? | |
| Should these also be part of nursing staff’s EOL conversations? | Could you please explain why? | |
| **To what extent do you find the framework applicable within each setting (i.e., hospital, nursing home, home)?** |  |  |
| **To what extent do you find the framework applicable to nursing staff of any level?** |  |  |
| **Points of discussion** | | |
| **How do you prepare yourself for an EOL conversation?** | Can/would you please explain why/how you do/how you deal with this? | |
| **How do you prepare the person you are having this conversation with for an EOL conversation?** |  |  |
| How do you handle your own emotions during a conversation? |  |  |
| Do you experience differences between formal (pre-planned) and informal (spontaneous) communication? |  |  |
| **What are your thoughts about the differences we found in the perspectives of older people and their family caregivers and nursing staff?**   - **Different expectations.** - **Suggestion that for these conversations, nursing staff should occasionally let go of control and instead focus on listening, being present and moving with them.** - **Usually no preparation by and few expectations from older people and their family caregivers combined with a lack of encouragement for this from nursing staff versus expecting a proactive role from them in ACP.** | Could you please explain why? | |
| What tools do you use when having EOL conversations? |  |  |
| **How would you describe interprofessional collaboration with nursing staff when it comes to EOL communication? What do you consider important in this?** | To what extent does this differ for nurses, care assistants and nurse practitioners? | |
| **Expertise professionals** | | |
| **What can you learn from nursing staff in EOL communication?** | | Could you please explain why? |
| **What can nursing staff learn from you regarding EOL communication?** | |  |
| **Closing** | | |
| Questions/reflection | | |

Part 3: Patient and family caregiver representatives

| **Opening question** | |
| --- | --- |
| **From your expertise, when is a conversation a good conversation?** | |
| What words come to mind when you think about the role of nursing staff EOL communication? | |
| **Overarching questions regarding the framework** | |
| **Looking at the framework as a whole, what thoughts come to mind?** | |
| **Are all parts of the framework clear to you?** | |
| **Are there parts that you miss in the framework?** | What components are you missing? Could you please explain why? |
| **Should these also be part of nursing staff’s EOL conversations?** | Could you please explain why? |
| **Points of discussion** | |
| Do you think that nursing staff should always engage in EOL communication themselves? | Could you please explain why? |
| **How do you think nursing staff should prepare for an EOL conversation?** |  |
| **How do you think nursing staff should prepare older people and their family caregivers for an EOL conversation?** |  |
| How do think nursing staff should handle their own emotions during an EOL conversation? |  |
| Do you think the framework should distinguish between formal (pre-planned) and informal (spontaneous) communication? |  |
| **What are your thoughts about the differences we found in the perspectives of older people and their family caregivers and nursing staff?**   - **Different expectations.** - **Suggestion that for these conversations, nursing staff should occasionally let go of control and instead focus on listening, being present and moving with them.** - **Usually, no preparation by and few expectations from older people and their family caregivers combined with a lack of encouragement for this from nursing staff versus expecting a proactive role from them in ACP.** |  |
| What do you think of the use of tools in EOL communication? |  |
| **How would you describe interprofessional collaboration as it relates to EOL communication? What do you consider important in this?** |  |
| **Closing** | |
| Questions/reflection | |

Part 4: Heterogenous focus group

| **Opening questions** | | |
| --- | --- | --- |
| **Looking at the framework as a whole, what thoughts come to mind?** | | |
| **Are all parts of the framework clear to you?** | | |
| **Short presentation of the framework with emphasis on the suggested changes in the fundamentals as a result of the previous focus groups & discussion.** | | |
| **Overarching questions regarding the framework** | | |
| **What do you think of the changes we made based on the initial focus groups?** | Could you please explain why? | |
| **Are there parts that you miss in the framework?** | What components are you missing? Could you please explain why? | |
| **Should these also be part of nursing staff’s EOL conversations?** | Could you please explain why? | |
| To what extent do you find the framework applicable within each setting (i.e., hospital, nursing home, home)? |  |  |
| To what extent do you find the framework applicable to nursing staff of any level? |  |  |
| **Points of discussion** | | |
| **What do you think of the suggestion that for EOL conversations, nursing staff should occasionally let go of control and the purpose of the conversation and instead focus on listening, being present and moving along?** | Could you please explain why? | |
| **Expertise professionals** | | |
| **What can you learn from nursing staff in EOL communication?** | | Could you please explain why? |
| **What can nursing staff learn from you regarding EOL communication?** | |  |
| **What would you like to advise us regarding the further development of the framework?** | |  |
| **Closing** | | |
| Questions/reflection | | |
