# Supplementary material for: Conceptualizing end-of-life communication by nursing staff as part of advance care planning with older people: A multiple discipline focus group study
Source: Int J Nurs Stud Adv. 2025 Oct 25;9:100436. doi: 10.1016/j.ijnsa.2025.100436 (PMC12603746; doi:10.1016/j.ijnsa.2025.100436)
Supplement: Supplementary file 3 [file mmc3.docx]

Appendix C: Coding framework

| **Theme** | **Fundamental** | **Code** |
| --- | --- | --- |
| Feeling comfortable (feeling seen and heard) | Being able to talk about the EOL | Being able to talk about the EOL personally  Being able to talk about the EOL professionally  Being aware of the presence or absence of this competency  This competency helps in being aware of and responding to cues from older people to start a conversation  Being aware of own values, beliefs and cultural background and how they affect the conversation  Showing emotions (appropriate/not appropriate) |
|  | Establishing a trusting relationship (feeling a human connection) | Relationship promotes interpretation of observations  Relationship promotes seeing the older person as a human being  Relationship promotes open and intimate conversations  Relationship promotes feeling a mutual click/connection  First impression can already determine relationship  Humor contributes to relationship building  Speaking dialect contributes to relationship building  Working experience contributes to relationship building  Exuding self-confidence contributes to relationship building  Older age contributes to relationship building through life experience  Spending a lot of time with the older person contributes to relationship building  Intimate moments contribute to relationship building  Relationship slowly strengthens as more conversations follow  Openness by older person suggests stronger relationship  Sharing emotions by older person suggests stronger relationship  Showing interest by older person suggests stronger relationship  Initiating conversations by older person suggests stronger relationship  Transfer conversations to colleague if a bond/relationship does not develop  Being able to see nurses as human beings  Nurses should be able to be themselves and share something about themselves  Humanity of nurses makes the conversation more open  Humanity of nurses allows older person to be more themselves |
|  | Learning by doing (readiness) | Less experienced nurses attach particular importance to interview techniques and awareness of important interview topics  More experienced nurses value knowledge  To feel comfortable in conversation, learning by doing is essential  With enough experience, EOL communication can be approached like any other person-centered conversation  EOL conversation should be about life, not just the end of life  Previous personal experiences with EOL or EOL communication contribute to being “ready” for EOL communication  Previous experiences with EOL communication make conversations easier because you can move more quickly to “heavier” topics  The earlier EOL communication occurs, the earlier older people begin thinking about future care  The sooner EOL communication occurs, the sooner older people become aware of the importance of these conversations |
|  | Having a natural and open conversation | Older people should be pain-free  Older people should feel safe  Observe whether older people feel comfortable enough to express themselves  Conversations that are/feel natural, calm/relaxed and open are particularly valued  Natural/quiet/open conversations make older people and family caregivers feel safe and at ease and share more information  Heavy topics should be discussed in a light-hearted way if possible  Humor is allowed in a conversation; contributes to a relaxed atmosphere that makes older people and family caregivers feel more at ease |
| Creating space for open communication (feeling seen and heard) | Moving along with the older person | Every EOL conversation is different; no fixed approach  Adapt approach to personality  Adapt approach to medical history  Adapt approach to prognosis  Adapt approach to life experience  Adapt approach to anxiety  Adapt approach to cognitive function  Adapt approach to cultural background  Adapt approach to emotional state  Adapt approach to physical constitution  Adapt approach to degree of openness/resistance  Adapt approach to facial expression  Adapt approach to body language  Adapt communication approach to atmosphere  Palliative care dimensions as the only fixed structure  Adapt approach conversation to the needs and preferences of the older person |
|  | Being easily approachable (valuing nursing staff’s attitude) | Showing empathy by listening attentively  Showing empathy by being attentive to the conversation  Showing empathy by not judging  Showing empathy by not imposing one's own views  Empathy by acknowledging the older person  Showing compassion by being close to the older person  Showing compassion by putting a hand on a shoulder  Showing compassion by holding a hand  Showing compassion by hugging  Showing compassion by calling older person by first name  Showing compassion by sitting next to someone  Showing compassion by giving the older person space to share their story  Showing compassion by adjusting own verbal tone  Being approachable by keeping conversations informal  Being approachable by having a welcoming appearance  Reach out by having an open attitude (do not cross arms, turn away)  Reachable by making eye contact  Share something about yourself in the conversation  It is important to approach the conversation personally, fluently and in a private rather than businesslike and therefore distant manner  Friendliness  Empathy  Caring  Speaking up  Non-judgmental/accepting  Understanding  Humanity  Nurse as professional (is close by, always ready, speaks the same language, has your best interests at heart, can talk to you about anything)  Transcending professionals; know everything about you and protect/defend you in interdisciplinary teams |
|  | Creating a calm atmosphere (valuing nursing staff’s attitude) | Taking your time  Show that you have plenty of time by speaking quietly  Minimize the number of people in the room  Closing the bed curtain  Minimizing distractions  Telling co-workers that you will be speaking to avoid interruption  Turning off devices that may be distracting  Relaxed attitude/atmosphere |
|  | Taking a seat (valuing nursing staff’s attitude) | Taking a seat shows that you see yourself as equal  Sitting down on a chair  Going to sit on the older person's bed if he/she is emotional  Sitting down strategically so that the older person does not have to keep turning around (if more than one person is present)  Sitting down is seen as a way to make the conversation feel more relaxed |
|  | Being honest (valuing nursing staff’s attitude) | Honest and direct communication to emphasize reliability  Honest and direct communication because the older person sometimes needs it  Direct communication in case of urgency  Reliability  Honesty  Being direct when necessary |
| Using senses and applying communication techniques (feeling seen and heard) | Listening (feeling heard) | Actively listen and understand what the older person is saying prioritize vs. anticipate the next question  Focusing on the older person  Listening to what is important to someone  Listening to what someone is concerned about  Listening to what information is important to ACP  Filtering by being alert to topics that are important to the older person  Filter to be able to deepen the conversation (ask more questions)  Remembering information to come back to at a later time to show that you have listened |
|  | Seeing | Attention to intonation and nonverbal communication (body language, facial expression, look in eyes)  Observe to recognize agitation, fear, sadness, misunderstanding, resistance  Observe consistency between verbal and nonverbal communication  Observe/signal changes in nonverbal expressions  If a person is not ready for EOL communication, he/she shows evasive body language, avoids eye contact, gives few/short/long/atypical responses, changes the subject, is agitated, or says they do not want to have the conversation  Speaking observations to avoid misinterpretation (asking for confirmation) and make someone aware of this |
|  | Speaking | Using plain language  Taking frequent pauses between sentences  Avoiding difficult words  Making short sentences  Explain information in a short and easy way with examples  Use the teach-back method to prevent misconception  Leaving regular silences so the older person can think/process  Speaking quietly  Do not ask too many questions; this hinders the flow of conversation |
|  | Intuition (feeling seen) | Making many decisions based on feeling/intuition  Approach to EOL communication is often intangible  Do not make EOL communication too theoretical  Older people and their family caregivers feel seen when the nurse senses when something is wrong or what the conversation should be about |
| Following the conversational phases (preparation and reflection) | Expectations | Few/no expectations for EOL conversations  Starting the conversation openly  Most important to be treated and seen as a person and appreciated in a positive conversation |
|  | Preparing the conversation | Being aware of earlier EOL conversations  Being aware of the reason for an EOL conversation  Being aware of who the older person is  Knowing the emotional state of the older person  Prepare by going through the patient file  Prepare by verbal handover from colleague  Complete preparation is usually not possible/not sufficient due to the dynamic nature of the conversations and the dependency of the older person  Preparation can lead to a limited view of the older person  Little preparation before an EOL conversation  Unaware of the purpose of the conversation (sometimes satisfactory, sometimes not)  Preparation by an information letter/folder, questionnaire or by writing down questions before the interview |
|  | Initiating the conversation | Initiate informal conversation by responding to cues  Initiate informal conversation spontaneously  Initiate formal conversation in response to clinical deterioration or admission to a nursing home  Initiate formal conversation by asking about current situation  Anticipate what the older person says first and then work toward the trigger or purpose of the conversation |
|  | Gently building up the conversation (readiness) | Approach and structure for the conversation depend on the older person's signals  Start conversation lightly and slowly build up to heavier topics  Speed of building up conversation depends on (the resistance of) the older person  Probe questions provides depth  If appropriate, scenarios are discussed  Emphasize that decisions do not have to be made immediately  Emphasize that decisions can always be reviewed  Find balance between gathering information to provide good care, informing and letting people vent  Content of conversation depends on the older person  Being ready for conversation promotes conversation management  Nurse adjusts approach based on whether (to what extent) older person is ready for EOL conversation |
|  | Evaluating (reflection) and following up the conversation | Asking if the older person has any questions  Asking if the older person missed anything in the conversation  Ask how the older person experienced the conversation  Evaluate later if the conversation has been heavy/difficult to give the older person space to calm down  At the end of the conversation ask if the older person would like a follow-up conversation  Follow-up conversation usually takes place on another day  Registration in the patient file always takes place after the conversation (appointments, topics of conversation, evaluation)  Older people and family caregivers reflect together on EOL conversation afterwards  EOL communication encourages (the importance of) reflecting together with family caregiver on treatment wishes  Receiving a summary of the conversation is a useful tool to fall back on and use for reflection.  Follow-up conversations are appreciated; this way older people and family caregivers feel seen and heard; they are not forgotten |
| Interprofessional collaboration (paying attention to family caregivers) | Perceiving your own professional role | Difficult to describe role (both individually and on the interdisciplinary team)  Some nurses have never thought about their role  Care organization often does not have agreements on roles/responsibilities in ACP  Nurse coaches  Nurse empowers  Nurse informs  Nurse makes people think  Nurse helps to structure the older person's thoughts.  Nurse finds it important to support and encourage colleagues in EOL communication  Nurse practitioner mostly engages in formal conversations  Nurse engages in both formal and informal conversations, but informal conversations are more common  Fundamentals are the same in formal and informal conversations; only the occasion, triggers, setting and participants often differ  Nurse practitioner puts more emphasis on treatment needs  Care assistant feels they are not allowed to interfere in conversations about treatment needs  Care assistant usually talks about what is important to the older person now and in the future and how he/she is feeling  Nurse practitioner and care assistant usually participate in conversation individually  Nurse sometimes participates in conversation together with physician  Home care nurse looks further ahead in conversation  Home care nurse places more emphasis on taking the lead regarding the provision of interprofessional support |
|  | Involving family caregivers (valuing family caregivers’ presence & considering family caregivers’ well-being) | Family caregivers should be involved in EOL communication (especially in formal conversations)  Involve family caregivers to provide additional information  Involve family caregivers to ask them for their opinion  Engaging family caregivers to let them vent  Involve family caregivers to have concerns expressed  Older person should always be in the lead  Help older people to be in the lead by asking questions directly to them and giving them the last word  Nurses minimize involvement in family conflicts  If necessary (in case of conflict), communicate only with the older person's first contact person  Involve family caregivers to inform  Involve family caregivers to agree  Involve family caregivers to complement the older person  Involve family caregivers to support subsequent reflection with the older person  Attention should be paid to (and actively asked about) the well-being of family caregivers |
|  | Involving colleagues | Nurses are curious about their colleagues' opinions  Nurses want to know if colleagues feel they have conducted the interview appropriately  Nurses want to reflect on the interview with colleagues  Nurses sometimes want to discuss the interview with a colleague (e.g. in the case of known resistance). |

Blue: Initially according to nursing staff

Purple: Initially according to older people and family caregivers

Green: Initially according to nursing staff & older people & family caregivers
